# Synthesis and photooxygenation of 3-(*p*-substituted phenyl)-3a,8a-dihydro-*4H*-cyclohepta[d]isoxazoles: facial selectivity

**DOI:** 10.55730/1300-0527.3688

**Published:** 2024-05-28

**Authors:** Mahire Emel OLGUN, Abdullah MENZEK, Ertan ŞAHİN, Yasin ÇETİNKAYA

**Affiliations:** 1 Department of Chemistry, Faculty of Science, Atatürk University, Erzurum, Turkiye; 2 Department of Emergency Aid and Disaster Management, Faculty of Health Sciences, Ardahan University, Ardahan, Turkiye

**Keywords:** Diels-Alder reaction, facial selectivity, isoxazole, photooxygenation, density functional theory

## Abstract

Two 3-(*p*-substituted phenyl)-3a,8a-dihydro-4*H*-cyclohepta[d]isoxazoles were synthesized by 1,3-dipolar cycloaddition of the corresponding nitrile oxides with cycloheptatriene. Two endoperoxides were synthesized as facially selective and single products in high yields (93%–95%) from the reactions of isoxazole derivatives with singlet oxygen. The exact configurations of the endoperoxide with a methyl group in the phenyl ring and the diol synthesized from it were confirmed by X-ray analysis. To elucidate the mechanism, the formation energy of the endoperoxide was investigated by simulations using the software package Gaussian 09 and density functional theory calculations via the M06-2X/6-311+G(d,p) level method in dichloromethane. The results were consistent with experimental findings showing the formation of isoxazole products.

## 1. Introduction

**Figure 1:**
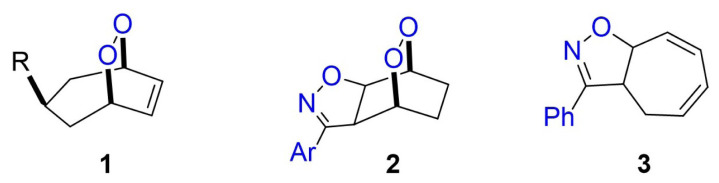
Important diene and endoperoxides.

Molecules containing halogen and oxygen derivatives such as hydroxyl, ether, peroxide, and epoxide are important because they can be considered target and intermediate products. Peroxide groups in molecules are generally obtained as unsaturated endoperoxides as a result of cycloaddition reactions of dienes with singlet oxygen [1,2]. The groups in the rings of unsaturated endoperoxides in the structure **1**, where R is Cl and OSiC(CH_3_)_3_, are in *cis-*configuration, and these endoperoxides are formed as the main product in the reactions of the relevant dienes with singlet oxygen [3,4]. The double bonds in the [2.2.1] and [2.2.2] skeleton structures are stressed due to pyramidazation and therefore show dienophile properties in cycloaddition reactions [5–8]. Endoperoxides in the structure **2**, *endo* and *exo*, were formed by the 1,3-dipolar cycloaddition of the corresponding unsaturated endoperoxide and nitrile oxide [9]. The known compound **3** [10] is a bicyclic isoxazole compound and has a conjugated diene structure in its seven-membered ring (Figure 1).

Compound **3** or its derivatives, having isoxazole and cycloheptadiene rings, can be photooxidized. Selectivity can be observed in the formation of these unsaturated endoperoxides and these products will contain three oxygen atoms and one nitrogen atom. Additionally, these products can also be converted into derivatives such as diols. Therefore, these products and reactions are important. For this reason, compounds with Me and OMe were synthesized in the para position of the phenyl ring in the compound **3** structure, and their photooxygenation reactions were examined. It was observed that endoperoxides were formed as single products in high yields in the reaction of isoxazole derivatives with singlet oxygen. To determine the configuration and formation of adducts, some computational studies (e.g., density functional theory (DFT)) were performed.

## 2. Results and discussion

### 2.1. Synthesis

**Scheme 1:**
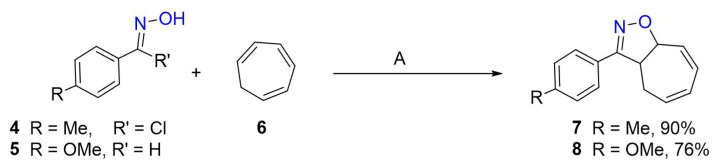
Synthesis of compounds **7** and **8**. A (NEt_3_, CH_2_Cl_2_, −5 °C to RT, 2 days for **7**), (Cold aqueous NaOCl, CH_2_Cl_2_, −5 °C to RT, 3 days for **8**).

As described previously [7,11,12], oximes **4** and **5** were synthesized to obtain new compounds that are derivatives of compound **3**. Therefore, the reactions of oximes **4** and **5** with cycloheptatriene (CHT) were carried out separately (Scheme 1). The products **7** and **8** obtained from each reaction were purified by column chromatography and obtained in high yields. Adducts **7** and **8** are structures in which five- and seven-membered rings are interlocked with each other. According to the ^1^H-NMR spectra of these products, the double bonds in the seven-membered rings were conjugated.

**Scheme 2:**
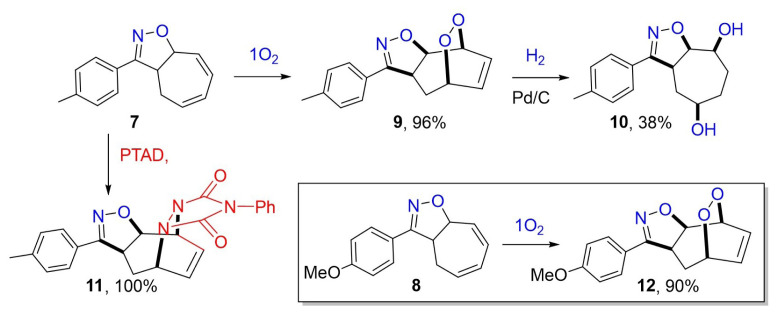
Reactions of compounds **7** and **8**.

Reactions such as Diels–Alder reactions can be performed with the conjugated diene in the seven-membered ring of compounds **7** and **8** and compounds with various functional groups, especially in its seven-membered ring, can be obtained. As a result of Diels–Alder reactions of conjugated dienes with singlet oxygen and PTAD (4-phenyl-1,2,4-triazoline-3,5-dione), oxygen and nitrogen atoms are placed at the 1,4 positions of the diene [1,2,13,14]. Compound **7** was reacted with both singlet oxygen and PTAD. Only one product from each of these reactions was isolated in high yield (Scheme 2). Since it is understood from their NMR spectra that there is only a double bond in their seven-membered ring, these products are adducts formed by Diels–Alder reactions.

A catalytic hydrogenation reaction is used in the reduction of many functional groups such as unsaturated endoperoxides [1,15–17]. The adduct, endoperoxide **9**, was reacted with gaseous hydrogen (approximately 1.0 atm) in the presence of catalyst Pd/C at room temperature (RT) (Scheme 2). A product was obtained from the purification process of the reaction mixture on the silica gel column chromatography. This product should be a diol derivative because no double bond was present in its seven-membered ring when its NMR spectra were investigated and it was more polar than the corresponding endoperoxide. X-ray analysis of the diol derivative both explains its configuration as **10** and suggests that the structures of the adducts formed from the reactions of **7** with singlet oxygen and PTAD should be **9** and **11**, respectively. Moreover, the proposed structure of endoperoxide **9** was confirmed by X-ray analysis of it.

**Figure 2:**
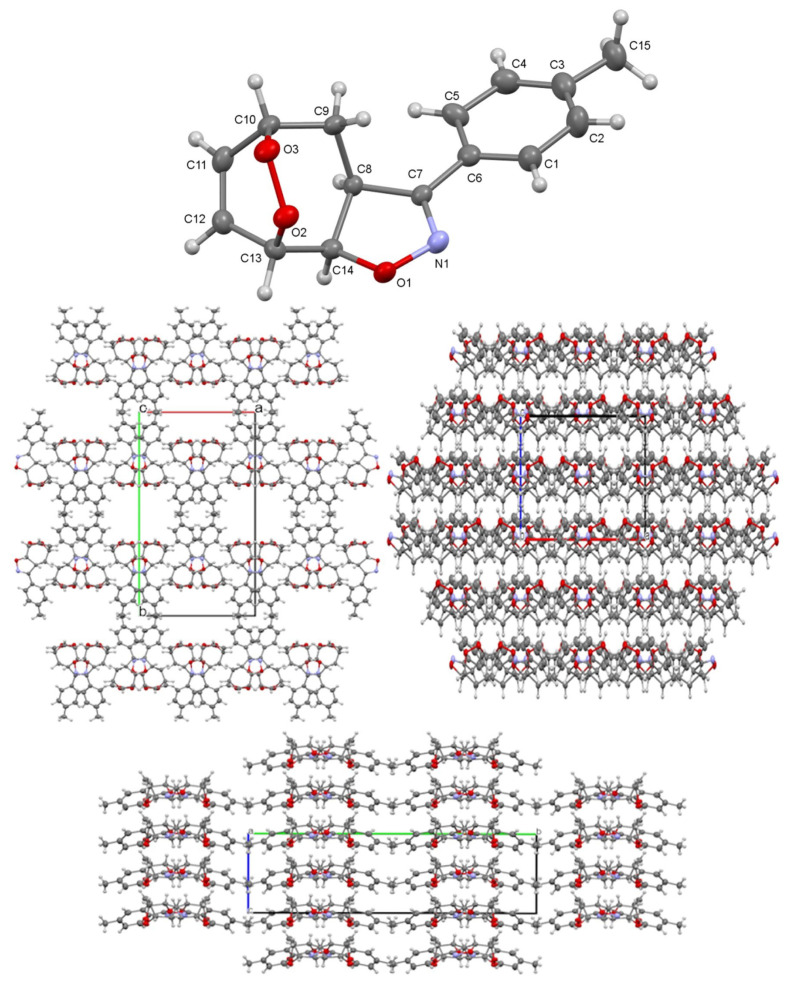
(Top) X-ray structure of the molecule **9**. Thermal ellipsoids are drawn at the 40% probability level. (Bottom) The crystal lattice and the unit cell viewed down along the *c*-, *b*-, and *a*-axis, respectively, with the square void motif.

**Figure 3:**
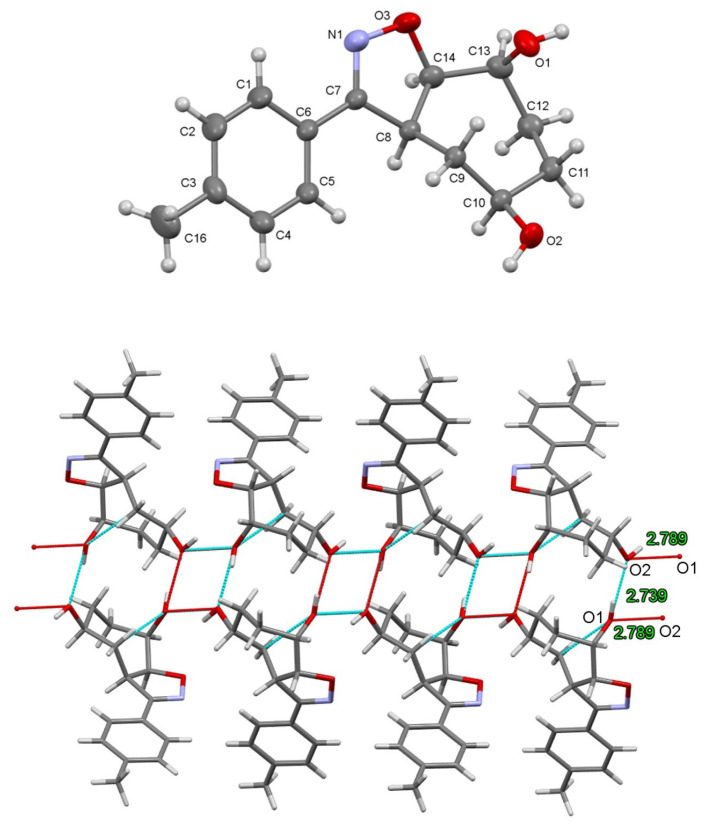
(Top) Molecular structure of **10**. Thermal ellipsoids are drawn at the 40% probability level. (Bottom) Consecutive tetramer chain structure with *H*-bonding geometry.

The exact conformation and structures of (3aS*,5S*,8S*,8aR*)-3-(4-methoxyphenyl)-3a,5,8,8a-tetrahydro-4H-5,8-epidioxycyclohepta[d]isoxazole (**9**) and (3aS*,5R*,8S*,8aR*)-3-(p-tolyl)-3a,5,6,7,8,8a-hexahydro-4H-cyclohepta[d]isoxazole-5,8-diol (**10**) were confirmed by X-ray diffraction analysis. Both solved molecules are in racemic form, and only one enantiomer is seen in the asymmetric unit (Figures 2 and 3). Molecule **9** was crystallized in the orthorhombic Iba2 space group. It consists of fused cycloheptene and heterocyclic oxazole rings with toluene and endoperoxide moiety. Here the cycloheptene ring is in the boat conformation and stressed by the endoperoxide. In the cycloheptene cycle, the length of the C11–C12 double bond is 1.333(3) Å, while the single bonds in the rest of the ring are in the range of 1.483–1.557(3) Å. Moreover, the O2–O3 length of peroxide is 1.461(3) Å and the N1-C7 double bond in the oxazole ring is 1.293(3) Å. For this structure, there is a C13-H···O2 interaction [*D*···A=3.168(3) Å] between consecutive enantiomers. The racemic nature of the structures significantly affects the supramolecular structures and crystal lattice motifs (Figure 2).

The other molecule **10** crystallizes in the monoclinic space group P2_1_/c with four molecules in the unit cell. The molecule consists of fused cycloheptane and heterocyclic oxazole rings with diol and toluene units. The seven-membered cycloheptane ring adopts a twist-chair conformation. The dihedral angle between the mean planes of the cycloheptane (C8/C14/C10/C11) and oxazole (O3/N1/C7/C8) rings is 29.8°. The C-C (cycloheptane) bond lengths are in the range of 1.516(3)–1.533(3) Å; all have a single bond character. Diol C1-O1 and C2-O2 single bonds are 1.433 Å and 1.420 Å in length, respectively. The N=C double bond in the oxazole ring is quite short (1.278 Å). The molecule forms a consecutive tetramer chain structure with O1-H···O2 [*D*···A=2.739(3) Å] and O2-H···O1 [*D*···A=2.789(3) Å] hydrogen bonds (Figure 3). The C-H-π interaction [X···Cg 3.691 Å, *Cg*; Center of gravity of C1/C6 phenyl ring] can be regarded as an important intermolecular interaction in the structure.

Furthermore, the reaction of compound **8** with singlet oxygen was performed (Scheme 2). The formation and configuration of endoperoxide **12** formed in this reaction should be very similar to those of endoperoxide **9** because it was also obtained in high yield in its reaction alone. It was accepted that the configuration of **12** was the same as that of **9**.

### 2.2. DFT calculations

As mentioned above, the formation of a single product in the Diels–Alder reactions of compounds **7** and **8** with dienophiles indicated that there was facial selectivity in them during these reactions. We also focused on testing this selectivity with a theoretical study and examined the mechanism in detail. In our previous research, we used quantum chemical calculations including DFT in the M06-2X method to examine the structural properties of compounds and shed light on the formation mechanism of reactions and obtained quite consistent results [18–20]. The fact that the method used in these studies yielded good results prompted us to use the same method in the quantum chemical calculations in the present study.

#### 2.2.1. Computational details

The DFT studies were carried out with the software package Gaussian 09 [21]. The visualizations of the geometric optimizations for all calculations were generated with the program CYLview v1.0.561 BETA [22] and the software GaussView 5.0 [23]. All calculations were performed using DFT with the M06-2X method at the 6-311+G(d,p) level in DCM, and the default polarizable continuum model (PCM) method was used [24,25]. In the present study, we examined the reaction mechanism by optimizing the product (endoperoxide) and the reactant obtained from the intrinsic reaction coordinate (IRC) calculation of the transition state (TS).

#### 2.2.2. Mechanistic calculations

**Scheme 3:**
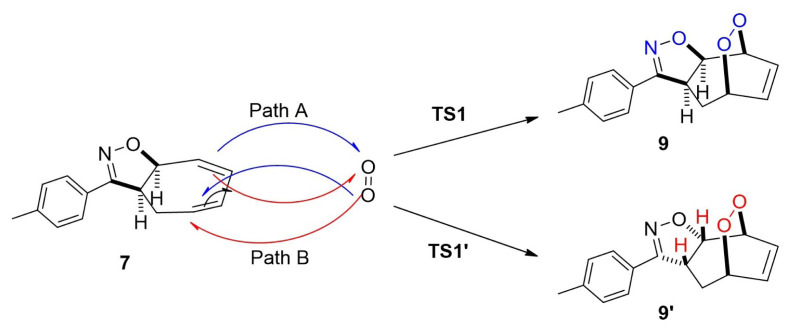
Paths A and B of the proposed mechanism for the endoperoxides.

There are two possible pathways for the [4+2] cycloaddition mechanism of ^1^O_2_ to 3-(p-tolyl)-3a,8a-dihydro-4H-cyclohepta[d]isoxazole (**7**), as shown in Scheme 3. We observed that the endoperoxides (**9** and **9′**) were formed when the products from IRC calculations of the TSs were optimized. In both TSs, the endoperoxide formation reaction occurred in an asynchronous concerted manner by the electrophilic attack of O2 on C1 and C4 on O1 with singlet oxygen approaching compound **7** from the same (path B) or opposite (path A) direction as H1 and H2.

**Figure 4:**
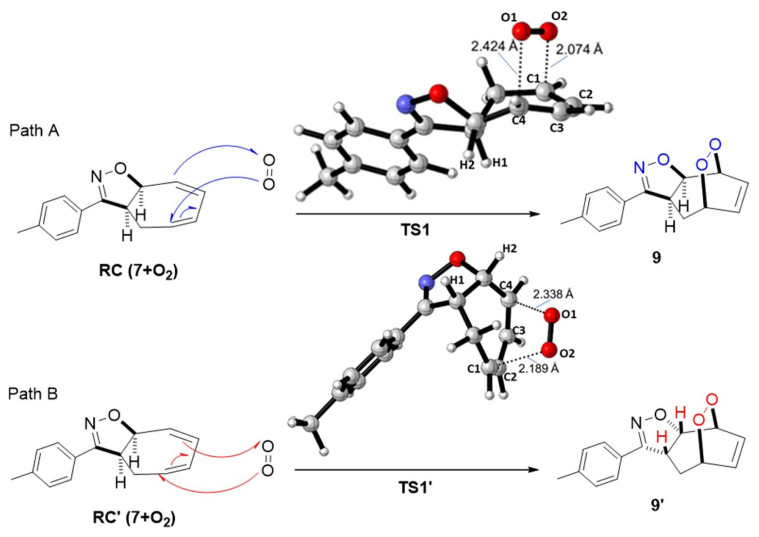
Possible paths A and B for the formation of the transition states TS1 and TS1′ for **9** and **9′**. Optimized transition structures at PCM/M06-2X/6-311+G(d,p)//M06-2X/6-311+G(d,p) level in DCM.

The first transition state (TS1) corresponds to the electrophilic attack of the singlet oxygen molecule (^1^O_2_) on the carbon atom (C1) in the less sterically face of diene **7** from the opposite direction as H1 and H2. This transition state is shown as path A in Scheme 3 and Figure 4. In the case of path B, the alternative transition state (TS1**′**) corresponds to the electrophilic attack of O2 on the C1 atom from the same direction as H1 and H2. The optimized geometries of the transition structures for paths A and B are shown in Figure 4.

**Figure 5:**
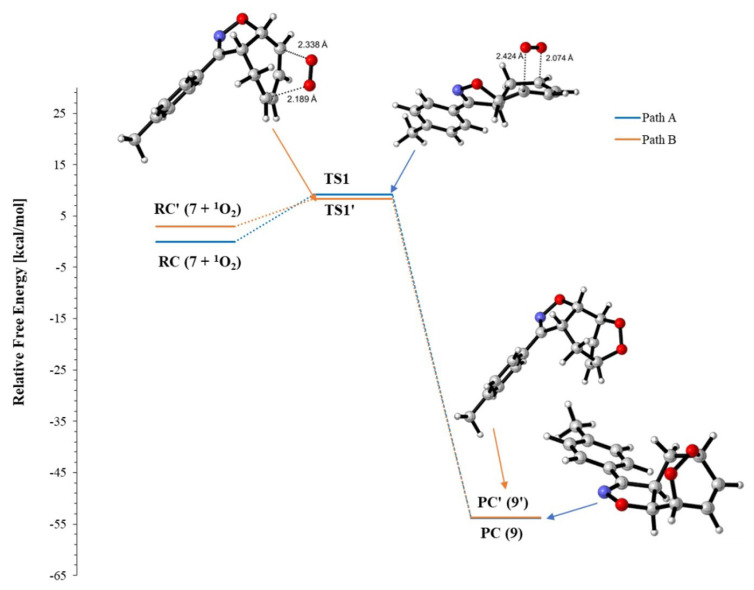
Relative Gibbs free energies (kcal/mol) for the possible paths A and B related to the formation of endoperoxide **9** at PCM/M06-2X/6-311+G(d,p)//M06-2X/6-311+G(d,p) level in DCM.

The relative Gibbs free energies of both mechanisms are presented in Table. While the TS1′ energy barrier of path B (8.3 kcal/mol) is lower than the TS1 energy barrier of path A (9.1 kcal/mol), the relative energy value of the reactant complex (**RC′**) for path B is higher by 2.9 kcal/mol than the energy of the reactant complex (**RC**) for path A. This result shows that **RC** is more stable than **RC′**. Considering the stability of the products obtained from both pathways, it was calculated that the relative Gibbs free energy of product **9** (product compound, **PC**) was lower by 0.2 kcal/mol than that of product **9′ (PC′)**. According to these results, since the relative energy values of the reactant complex (**RC**) and product (**PC**) of path A are lower than those of path B (**RC′** and **PC′**), the product to be formed is expected to be obtained from path A. For paths A and B, potential energy profiles in DCM are depicted in Figure 5. The TS1**′** energy barrier of path B is lower by 0.8 kcal/mol than the TS1 energy value of path A. However, this value represents a very small energy difference for the transition state during the formation of the product. Here the most important driving force in the formation of the product is the relative energy values of the initially formed reactant complex.

## 3. Conclusion

As seen in Scheme 1, two new isoxazole derivatives (**7** and **8**) were synthesized from the reactions of CHT with oximes **4** and **5**. It was observed that the endoperoxides **9** and **12** obtained during their reactions were formed as single products in high yields by the Diels–Alder reaction. Compound **7** was also reacted with PTAD and the adduct **11** was obtained quantitatively from this reaction. Diol **10** was obtained from the reaction of endoperoxide **9** with catalytic hydrogenation. To determine the configurations of endoperoxides (**9** and **12**) and interpret the formation of adducts, X-ray diffraction analyses of compounds **10** and **12** were performed, respectively. Furthermore, the analyses indicated that the observation of facial selectivity in Diels–Alder reactions is the result of the approach of the dienophiles (^1^O_2_ and PTAD) to the diene moieties of **9** and **10** from only one side. Facial selectivity in the adducts was also investigated theoretically, taking into account the formation of endoperoxide **9**. After the structure of compound **9** was elucidated by X-ray analysis, it was examined by the M06-2X/6-311+G(d,p) level method in DCM to understand the formation mechanism of the product. Our computational studies supported the experimental findings showing that benzoxazole products are formed in this reaction. This research will make significant contributions to the study of elucidating the structure of the products that will be formed during the photooxygenation of isoxazole derivatives in which the cyclic structures are adjacent.

## 4. Experimental

### 4.1. General

The chemicals (including solvents) used in the experiments were prepared by known methods. Oximes were synthesized as reported previously [11,13]. The data of the synthesized compounds such as NMR (400 MHz Varian and Bruker), TLC (including preparative TLC), and HRMS [LC–MS–TOF electrospray ionization (1200/6210, Agilent)] were obtained as explained previously [7,11,12,26–28]. All NMR and HRMS spectra are given in Supplementary Material.

### 4.2. Synthesis of 3-(*p*-tolyl)-3a,8a-dihydro-4*H*-cyclohepta[d]isoxazole (7)

A stirring solution of compound **4** (2.6 g, 1.0 equiv.) and **6** (5.0 equiv.) in CH_2_Cl_2_ (30 mL) was cooled in the ice bath and then a solution of NEt_3_ (2.98 g, 2.0 equiv.) in CH_2_Cl_2_ (10 mL) was added dropwise to this solution. After the mixture was stirred for 2 days without further bath cooling, the reaction mixture was monitored by TLC and the reaction was completed. Water (15 mL) was added to the mixture and then the pH of the mixture was adjusted to 6–7 with dilute HCl solution. The mixture was extracted with CH_2_Cl_2_ (2 × 15 mL) and after the organic phases were combined, the resulting solution was dried over Na_2_SO_4_, and the solvent was removed under low pressure. Compound **7** (3.13 g, 90%) was obtained from the purification of crude product on silica gel (45 g) column chromatography (CC) by EtOAc/petroleum ether (5/95) elution. Mp: 83–85 °C; ^1^H-NMR (400 MHz, CDCl_3_): 7.63 (d, *J* = 8.4 Hz, A part of AB system, 2H), 7.24 (d, *J* = 8.4 Hz, B part of AB system, 2H), 6.32 (dd, *J* = 11.6, 3.2 Hz, A part of AB system, 1H), 6.22–6.05 (m, 3H), 4.95 (dd, *J* = 8.5, 2.8 Hz, OCH, 1H), 3.40 (ddd, *J* = 9.6, 8.5, 2.2 Hz, CCH, 1H), 2.39 (s, CH_3_, 3H), 2.34–2.25 (m, 1H), 2.20–2.09 (m, 1H):^13^C NMR (100 MHz, CDCl_3_):160.94 (CN), 140.43 (C), 133.26 (CH), 129.60 (2 CH), 128.64 (CH), 127.79 (CH), 126.97 (2 CH), 126.81 (CH), 125.84 (C), 83.08 (OCH), 50.82 (CH), 27.01 (CH_2_), 21.63 (CH_3_); R_f_: 0.51 EtOAc/hexane (1/19); HRMS: (APCI – TOF) (m/z) calcd for [C_15_H_15_NO + H]^+^: 226.12319; found: 226.12249.

### 4.3. Synthesis of 3-(4-methoxyphenyl)-3a,8a-dihydro-4*H*-cyclohepta[d]isoxazole (8)

A stirring mixture of oxime **5** (5.0 g, 33.1 mmol, 1.0 equiv.), CHT (15.2 g, 5.0 equiv.), and CH_2_Cl_2_ (100 mL) was cooled with ice. Next, cold aqueous NaOCl (sodium hypochlorite) solution (5.25%, 45 mL, 1.072 g/mL) solution was added to the mixture, and then the mixture was stirred for 3 days without further bath cooling. After the organic phase of the mixture was separated and the aqueous phase was extracted with CH_2_Cl_2_ (2 × 30 mL), the combined organic phases were dried over Na_2_SO_4_ and the solvent was removed by rotary evaporator. The reaction mixture was subjected to CC on silica gel (55 g) and eluted using EtOAc/petroleum ether (by changing from 15/85 to 30/70). Product **8** (6.1 g, 76%) was obtained almost pure, and **8** was also obtained from this product pure using EtOAc/petroleum ether (30/70) by preparative TLC. Mp: 105–107 °C; ^1^H-NMR (400 MHz, CDCl_3_): 7.68 (d, *J* = 9.0 Hz, A part of AB system, 2H), 6.94 (d, *J* = 9.0 Hz, B part of AB system, 2H), 6.31 (dd, *J* = 11.6, 3.2 Hz, A part of AB system, 1H), 6.22–6.02 (m, 3H), 4.92 (dd, *J* = 8.4, 3.2 Hz, CHO, 1H), 3.85 (s, OMe, 3H), 3.37 (ddd, *J* = 17.2, 8.4, 2.0 Hz, CCH, 1H), 2.28 (bdd, *J* = 8.4, 3.2 Hz, A part of AB system, CH_2_, 1H), 2.19–2.08 (m, CH_2_, 1H); ^13^C-NMR (100 MHz, CDCl_3_): 161.30 (C), 160.86 (C), 133.49 (CH), 128.79 (CH), 128.76, 127.98 (2 CH), 126.99 (CH), 121.66 (CH), 114.56 (2 CH), 83.12 (OCH), 55.67, 51.25, 27.29 (CH_2_); R_f_: 0.54 EtOAc/hexane (1/5); HRMS (APCI – TOF) (m/z) calcd for [C_15_H_15_NO_2_ + H]^+^: 242.11810; found: 242.11699.

### 4.4. Photooxygenation of compound 7

A solution of compound **7** (650 mg) and catalytic tetraphenylporphyrin (TPP) in CH_2_Cl_2_ (90 mL) was placed in a flask continuously cooled by tap water on the outside. This solution was irradiated with a projector lamp (150 W) while a slow stream of dry O_2_ was passed through it continuously. The reaction was continuously checked by TLC, and after 6 days, the solvent was removed at room temperature in a rotary evaporator. The resulting endoperoxide **9** (713 mg, 96%) was crystallized by spontaneously removing the solvent from its concentrated solution in CH_2_Cl_2_ at RT. (3aS*,5S*,8S*,8aR*)-3-(*p*-tolyl)-3a,5,8,8a-tetrahydro-*4H*-5,8-epidioxycyclohepta[*d*]isoxazole (**9**): Mp: 157–159 °C; ^1^H-NMR (400 MHz, CCCl_3_): 7.53 (d, *J* = 7.8 Hz, A part of AB system, 2H), 7.21 (d, *J* = 7.8 Hz, B part of AB system, 2H), 6.74—6.67 (m, 1H), 6.58–6.52 (m, 1H), 5.15 (d, *J* = 5.9 OCH, 1H), 4.82 (dd, *J* = 9.6, 5.9 OCH, 1H), 4.76 (t, *J* = 5.9 OCH, 1H), 3.68–3.60 (m, CCH, 1H), 2.59–2.50 (m, CH_2_, 1H), 2.34 (s, CH_3_, 3H), 2.43–2.33 (m, CH_2_, 1H); ^13^C-NMR (100 MHz, CDCl_3_): 160.72 (CN), 140.57 (C), 139.90 (CH), 129.61 (2 CH), 127.10 (2 CH), 125.84 (C), 84.93 (OCH), 75.96 (OCH), 74.15 (OCH), 43.75(CH), 34.09 (CH_2_), 21.44 (CH_3_); R_f_: 0.41 EtOAc/hexane (2/3); HRMS: m/z (M+ 3H) calcd. For C_15_H_18_NO_3_: 260.12867; Found: 260.12882.

### 4.5. Catalytic hydrogenation of endoperoxide 9

After compound **9** (300 mg, 1.17 mmol), Pd/C catalyst (6 mg), and EtOAc (30 mL) were placed in a flask (100 mL, two-necked) fitted with a spin bar at RT, the air in the flask was replaced 3 times by hydrogen gas (about 1 atm in the balloon attached to the flask). Subsequently, the reaction was started, checked from time to time by TLC, and terminated after 5 days. The reaction mixture was filtered through filter paper to remove the catalyst and EtOAc was removed by rotary evaporator at RT. Diol **10** (115 mg, 38%) was obtained from the purification of residue on silica gel column chromatography (25 g) with EtOAc/petroleum ether (2/3) and crystallized in the mixture of CHCl_3_/hexane. (3aS*,5R*,8S*,8aR*)-3-(*p*-tolyl)-3a,5,6,7,8,8a-hexahydro-*4H*-cyclohepta[*d*]isoxazole-5,8-diol (**10**): Mp: 198–200 °C;^1^H NMR [400 MHz, (CD)_3_CO)]: 7.54 (d, *J* = 8.0 Hz, A part of AB system, 2H), 7.25 (d, *J* = 8.0 Hz, B part of AB system, 2H), 4.57 (dd, *J* = 11.6, 3.6 Hz, A part of AB system, 1H), 4.29–4.23 (m, 1H), 4.00–3.91 (m, 2H), 3.68–3.3.60 (m, 5H), 2.87 (3, 3H), 2.23–2.12 (m, 1H), 2.06–1.92 (m, 5H), 1.83–1.73 (m, 1H), 1.59–1.50 (m, 1H): ^13^C NMR [400 MHz, (CD)_3_CO)]: 159.49 (NC) 139.42 (C), 129.47 (2 CH), 127.27 (2 CH), 126.97 (C), 86.64 (OCH), 72.29 (OCH), 67.65 (OCH), 37.16, 32, 32.09, 26.31, 20.64; R_f_: 0.26 EtOAc/hexane (1/3); HRMS (APCI – TOF) (m/z) calcd for [(C_15_H_19_NO_3_) + H]+: 262.14432; found: 262.14353.

### 4.6. Cycloaddition reaction of compound 7 with PTAD

PTAD (80 mg, 0.46 mmol, 1.0 equiv.) was added to a solution of the compound **7** (103 mg) in CH_2_Cl_2_ (15 mL) at RT. The mixture was stirred for 1 day and it was observed that its red color changed to pale yellow. Adduct **11** (183 mg, quantitative) was obtained and crystallized from EtOAc. (3aS*,5S*,11S*,11aR*)-8-phenyl-3-(*p*-tolyl)-3a,4,11,11a-tetrahydro-*5H,7H*-5,11-ethenoisoxazolo[*5,4-d*][1,2,4]triazolo[*1,2-a*][1,2]diazepine-7,9(8*H*)-dione (**11**). Mp: 193–195 °C; ^1^H-NMR (400 MHz, CDCl_3_): 7.51–7.35 (m, 7H), 7.24 (d, *J* = 8.0 Hz, B part of AB system, 2H), 6.49 (dd, *J* = 8.8, 6.6 Hz, A part of AB system, 1H), 6.25 (ddd, *J* = 8.8, 6.6, 0,5 Hz, B part of AB system, 1H), 5.21–5.18 (m, NCH, 1H), 5.03 (dd, *J* = 9.8, 4.6 OCH, 1H), 4.94–4.91 (m, NCH, 1H), 4.25 (dt, *J* = 9.8, 4.0 CCH, 1H), 2.47 (ddd, *J* = 14.7, 9.2, 3.6 Hz, A part of AB system, CH_2_, 1H), 2.40 (s, CH_3_, 3H), 2.18 (dt, *J* = 14.7, 4.3 Hz, B part of AB system, CH_2_, 1H); ^13^C-NMR (100 MHz, CCCl_3_): 161.63 (CN), 151.78 (CO), 151.43 (CO), 140.85 (C), 131.49 (C), 129.99 (CH), 129.81 (2 CH), 129.20 (2 CH), 129.07 (CH), 128.34 (CH), 127.50 (2 CH), 126.56 (2 CH), 124.84 (C), 81.07 (OCH), 51.48 (NCH), 49.29 (NCH), 45.29 (CH), 27.75 (CH_2_), 21.23 (CH_3_); R_f_: 0.52 EtOAc/hexane (1/1); HRMS (APCI – TOF) (m/z) calcd for [(C_23_H_20_N_4_O_3_) + H]^+^: 401.16137; found: 401.16017.

### 4.7. Photooxygenation of compound 8

The reaction was carried out like the photooxygenation of compound **7** in 4.4. The reaction was realized according to procedure 1. Compound **8** (180 mg), CH_2_Cl_2_ (70 mL), and TPP (catalytic) were used in the reaction. The reaction lasted 3 days and its crystallization was carried out like that of endoperoxide **9**. (3aS*,5S*,8S*,8aR*)-3-(4-methoxyphenyl)-3a,5,8,8a-tetrahydro-*4H*-5,8-epidioxycyclohepta[*d*]isoxazole (**12**) (183 mg, 90%) was obtained from the reaction. Mp: 161–163 °C; ^1^H-NMR (400 MHz, CDCl_3_): ^1^H NMR (400 MHz, (CDCl_3_): 7.57 (d, *J* = 9.2 Hz, A part of AB system, 2H), 6.91 (d, *J* = 9.2 Hz, B part of AB system, 2H), 6.69 (dd, *J* = 9.1, 7.0 Hz, A part of AB system, 1H), 6.54 (ddd, *J* = 9.1, 7.0, 2.0 Hz, B part of AB system, 1H), 5.14 (dm, *J* = 6.4 Hz, NOCH, 1H), 4.82–4.73 (m, OOCH, 2H), 3.82 (OMe), 3.61 (dt, *J* = 10.0, 8.4 Hz CCHC, 1H), 2.55 (ddd, *J* = 15.2, 8.4, 5.8 Hz, CH_2_, 1H), 2.45 (ddd, *J* = 15.2, 10.4, 1.6 Hz, CH_2_, 1H); ^13^C-NMR (100 MHz, CDCl_3_): 161.38 (CN or CO), 160.65 (CN or CO), 134.15 (CH), 128.87 (2 CH), 127.35 (CH), 121.31 (C), 114.54 (2 CH), 85.09 (OCH), 86.19 (OCH), 85.09 (OCH), 74.27 (OCH), 55.62 (OCH_3_), 44.04 (CH), 34.35 (CH_2_); R_f_: 0.24 EtOAc/hexane (2/3); HRMS (APCI – TOF) (m/z) calcd for [C_15_H_15_NO_2_ + H]^+^: 274.10793; found: 274.10642.

### 4.8. Crystal structure determination

For the crystal structure determination, single crystals of the molecules of **9** and **10** were used for data collection on a four-circle Rigaku R-AXIS RAPID-S diffractometer (equipped with a two-dimensional area IP detector). Graphite-monochromated Mo-Kα radiation (l = 0.71073 Å) and oscillation scans with D*w* = 5° for one image were used for data collection. The lattice parameters were determined by the least-squares methods on the basis of all reflections with *F*^2^>2*s*(*F*^2^). Integration of the intensities, correction for Lorentz and polarization effects, and cell refinement were performed using the software CrystalClear (Rigaku/MSC Inc., 2005) [29]. The structures were solved by direct methods using SHELXS-2013 [30], which allowed location of most of the heaviest atoms, with the remaining nonhydrogen atoms being located from difference Fourier maps calculated from successive full-matrix least squares refinement cycles on *F*^2^ using SHELXL-2013 [30]. All nonhydrogen atoms were refined using anisotropic displacement parameters. The hydrogen atoms were assigned with common isotropic displacement factors and included in the final refinement using geometrical restrains. The final difference Fourier maps showed no peaks of chemical significance. *Crystal data for* **9**: C_15_H_15_O_3_N, crystal system, space group: orthorhombic, Iba2; (no: 45); unit cell dimensions: a = 13.3717(5), b = 26.251(2), c = 8.3998(3) Å, α = 90, β = 90, γ = 90°; volume; 2948.5(2) Å^3^, Z = 8; calculated density: 1.159 g/cm^3^; absorption coefficient: 0.663 mm^−1^; *F*(000): 1088; *θ*-range for data collection 3.3–74.9°; refinement method: full matrix least-square on *F*^2^; data/parameters: 2627/174; goodness-of-fit on *F*^2^: 1.108; final R-indices [I > 2s (I)]: R_1_ = 0.086, wR_2_ = 0.224; largest diff. peak and hole: 0.360 and −0.407 e Å^−3^. *Crystal data for* **10**: C_15_H_19_NO_3_, crystal system, space group: monoclinic, P2_1_/c; (no: 14); unit cell dimensions: a = 17.522(4), b = 6.6629(3), c = 11.695(2) Å, α = 90, β = 97.951(8), γ = 90°; volume; 1352.2(5) Å^3^, Z=4; calculated density: 1.284 g/cm^3^; absorption coefficient: 0.724 mm^−1^; *F*(000): 560; *θ*-range for data collection 2.5–79.4°; refinement method: full matrix least-square on *F*^2^; data/parameters: 2387/176; goodness-of-fit on *F*^2^: 1.085; final R-indices [I > 2s (I)]: R_1_ = 0.045, wR_2_ = 0.126; largest diff. peak and hole: 0.210 and −0.161 eÅ^−3^.

CCDC-2316861 (**9**) and 2320571 (**10**) numbers contain the supplementary crystallographic data. These data are provided free of charge via the joint CCDC/FIZ Karlsruhe deposition service: www.ccdc.cam.ac.uk/structures

## Supplementary Data

**Content**

Figures S1–S18 ^1^H, ^13^C NMR, and HRMS spectra of compounds **7–12**. Cartesian coordinates for the optimized structures.

Figure S1 ^1^H-NMR spectrum of **7** (400 MHz, CDCl_3_).

Figure S2 ^13^C-NMR spectrum of **7** (100 MHz, CDCl_3_).

Figure S3 ^1^H-NMR spectrum of **8** (400 MHz, CDCl_3_).

Figure S4 ^13^C-NMR Spectrum of **8** (100 MHz, CDCl_3_).

Figure S5 ^1^H-NMR spectrum of **9** (400 MHz, CDCl_3_).

Figure S6 ^13^C-NMR spectrum of **9** (100 MHz, CDCl_3_).

Figure S7 ^1^H-NMR spectrum of **10** [400 MHz, (CD)_3_CO)].

Figure S8 ^13^C-NMR spectrum of **10** [100 MHz, (CD)_3_CO)].

Figure S9 ^1^H-NMR spectrum of **11** (400 MHz, CDCl_3_).

Figure S10 ^13^C-NMR spectrum of **11** (100 MHz, CDCl_3_).

Figure S11 ^1^H-NMR spectrum of **12** (400 MHz, CDCl_3_).

Figure S12 ^13^C-NMR spectrum of **12** (100 MHz, CDCl_3_).

Figure S13 Compound **7**.

Figure S14 Compound **8**.

Figure S15 Compound **9**.

Figure S16 Compound **10**.

Figure S17 Compound **11**.

Figure S18 Compound **12**.

**Cartesian coordinates for the optimized structures (M06-2X/6-311+G(d,p)**

**Table:** Structure no.: TS1

	X	Y	Z
C	−3.38283900	−1.70266400	−0.15088600
C	−2.31430200	−1.76490500	0.72578200
C	−1.11463000	−0.87214800	0.79238600
C	−0.77838800	−0.13255800	−0.50552900
C	−1.78746700	0.93179200	−0.89341400
C	−3.25301500	0.68902500	−0.77711100
C	−3.86140400	−0.52994100	−0.78274200
C	0.38932500	0.79997500	−0.23667000
N	0.01271100	2.00195500	−0.01113500
O	−1.36955500	2.09183900	−0.15298800
C	1.79671000	0.39181700	−0.11586400
C	2.18740100	−0.90367700	−0.45330600
C	3.51904700	−1.29369100	−0.33510600
C	4.48956100	−0.40426400	0.11896300
C	4.08917600	0.89556600	0.45717300
C	2.76817100	1.29118200	0.34509800
C	5.93264000	−0.81217900	0.24739100
H	−4.05266300	−2.55542500	−0.15268400
H	−2.23783800	−2.68041700	1.30385300
H	−0.25609400	−1.47151300	1.10446300
H	−1.27309700	−0.11883000	1.57270200
H	−0.59802300	−0.82548000	−1.32785800
H	−3.85711700	1.55404600	−1.03644800
H	−4.90531200	−0.55690100	−1.08004700
H	1.45701000	−1.61859300	−0.81562800
H	3.80324900	−2.30539600	−0.60339900
H	4.82989700	1.60225800	0.81744100
H	2.47336600	2.29788100	0.61561800
H	6.56607800	−0.19464000	−0.39403900
H	6.28186900	−0.68086400	1.27414900
H	6.07541400	−1.85585800	−0.03302600
H	−1.62121200	1.17594700	−1.95581200
O	−3.45335700	0.56728400	1.63553500
O	−3.51440000	−0.62919100	1.97938900

**Table:** Structure no.: RC (7+ ^1^O_2_)

	X	Y	Z
C	−3.33418100	−1.39766700	−1.00301300
C	−2.16495400	−1.83119500	−0.50130600
C	−1.14489200	−0.94116500	0.13704300
C	−0.68334300	0.18011900	−0.81216400
C	−1.67056500	1.33478300	−0.92285300
C	−3.14060400	1.11269400	−0.79486300
C	−3.83276500	−0.03056300	−0.91915000
C	0.47082700	0.94177700	−0.18837100
N	0.10680100	2.04727500	0.33991300
O	−1.25234900	2.24111200	0.12856700
C	1.85269100	0.44810200	−0.08530300
C	2.24049300	−0.70679500	−0.76415200
C	3.54702500	−1.17912600	−0.66656000
C	4.49436100	−0.51424500	0.10767900
C	4.09718000	0.64431400	0.78821600
C	2.80088100	1.12054500	0.69787200
C	5.90960300	−1.01462800	0.22078700
H	−4.01731600	−2.12812300	−1.42453300
H	−1.93755400	−2.89043300	−0.56816100
H	−0.28522300	−1.52768500	0.46438200
H	−1.56669500	−0.45583100	1.02792500
H	−0.43551500	−0.22026200	−1.79527700
H	−3.70504300	2.03632200	−0.69878000
H	−4.91568900	0.06172100	−0.93351400
H	1.52741300	−1.24763800	−1.37623700
H	3.82946400	−2.07842500	−1.20298400
H	4.82008700	1.17444600	1.39976800
H	2.50836400	2.01492500	1.23470100
H	6.61345800	−0.27337300	−0.16537600
H	6.17118100	−1.20089700	1.26506600
H	6.04715100	−1.94064900	−0.33766000
H	−1.48689000	1.86201700	−1.87016500
O	−3.79761000	−0.31107200	1.94951600
O	−3.77070900	−1.49488300	1.77670200

**Table:** Structure no.: Compound 9 (PC)

	X	Y	Z
C	−3.25295500	−1.72887500	−0.60833100
C	−2.45377600	−1.64061300	0.66807800
C	−1.02737500	−1.08554900	0.51945100
C	−0.81557500	−0.06374800	−0.62257900
C	−1.89104200	1.00966600	−0.83850600
C	−3.28511000	0.66793300	−0.32349400
C	−3.74129500	−0.57411000	−1.04495700
C	0.34505500	0.85030700	−0.29297800
N	−0.01182100	2.04818200	−0.02785400
O	−1.38840400	2.17642100	−0.16795400
C	1.74807300	0.42118400	−0.17542100
C	2.16132700	−0.79422400	−0.72023100
C	3.48743800	−1.20422900	−0.60872600
C	4.42875300	−0.41647400	0.04935700
C	4.00615300	0.80268800	0.59512300
C	2.69009300	1.21807300	0.48878600
C	5.86349500	−0.85212700	0.18293200
H	−3.47037300	−2.68513600	−1.06785000
H	−2.42288800	−2.59934400	1.18750500
H	−0.34310500	−1.92051900	0.35662700
H	−0.76238100	−0.63910400	1.48164500
H	−0.64961300	−0.59829700	−1.55809300
H	−3.95756900	1.51109900	−0.48975400
H	−4.39566700	−0.47757800	−1.90211000
H	1.45311700	−1.42730900	−1.24297700
H	3.79106700	−2.15219300	−1.03955500
H	4.72390700	1.42776200	1.11641600
H	2.37474200	2.15885400	0.92375600
H	6.53184400	−0.13479800	−0.29937300
H	6.15278400	−0.91091100	1.23490200
H	6.02381300	−1.82975000	−0.27222600
H	−1.96020900	1.24679600	−1.90657600
O	−3.21935400	0.52710900	1.10979300
O	−3.21552600	−0.82431000	1.56586300

**Table:** Structure no.: TS1′

	X	Y	Z
C	−1.44345500	−1.32141400	1.31349200
C	−1.20076400	−1.77189000	0.04076800
C	−1.14919400	−0.99900300	−1.24028000
C	−1.06379600	0.51641100	−1.16509900
C	−2.22403700	1.25596400	−0.51617300
C	−2.73992900	0.74001300	0.80319300
C	−2.18066800	−0.15894000	1.65943200
C	0.06949500	1.15721400	−0.38206300
N	−0.26330200	2.28652800	0.11225700
O	−1.61350500	2.52970500	−0.17847200
C	1.42852600	0.62078700	−0.21286700
C	2.07015200	−0.04316000	−1.26055300
C	3.36058400	−0.53263900	−1.09391400
C	4.03547700	−0.38523400	0.11879000
C	3.38507200	0.28069300	1.16200500
C	2.09895500	0.77681200	1.00371400
C	5.41648900	−0.95310500	0.31184400
H	−1.24827600	−2.01893700	2.12083900
H	−0.84029500	−2.79287600	−0.04245800
H	−2.02521200	−1.26592700	−1.84028000
H	−0.28909500	−1.37328400	−1.80067700
H	−0.97109800	0.88806600	−2.19372100
H	−3.48705200	1.40784700	1.22367500
H	−2.52900800	−0.12511400	2.68730200
H	1.57688000	−0.15207300	−2.22018400
H	3.85219000	−1.03391100	−1.92087500
H	3.89306600	0.40705900	2.11241000
H	1.59898100	1.28092000	1.82265300
H	6.02838700	−0.29634500	0.93222200
H	5.36292100	−1.92410200	0.81213900
H	5.91974200	−1.09792600	−0.64470700
H	−3.04263800	1.46730700	−1.20137300
O	−3.36277200	−2.09397300	−0.07308200
O	−3.91545100	−0.99466400	−0.23425800

**Table:** Structure no.: RC′ (7+ ^1^O_2_)

	X	Y	Z
C	−1.57520400	−1.02424100	1.33010200
C	−0.97590200	−1.43727900	0.20112200
C	−1.19311400	−0.81145100	−1.14376900
C	−0.95338200	0.70239700	−1.17658500
C	−2.08503500	1.55375000	−0.60274100
C	−2.87650100	1.01151500	0.56136300
C	−2.59769400	0.01071900	1.41318400
C	0.19221200	1.24639300	−0.34568000
N	−0.07992300	2.36529300	0.20451300
O	−1.38958000	2.73189400	−0.10972900
C	1.51224300	0.61898300	−0.18627100
C	2.05072000	−0.16728200	−1.20658900
C	3.30658200	−0.74557900	−1.05857200
C	4.04811800	−0.56596700	0.10962500
C	3.49980200	0.22077700	1.12707200
C	2.24926300	0.80483100	0.98678600
C	5.39021100	−1.22637800	0.28281000
H	−1.36001500	−1.55427900	2.25203000
H	−0.28244600	−2.27011900	0.25931900
H	−2.23075500	−0.97889600	−1.46394300
H	−0.55958300	−1.30308700	−1.88113800
H	−0.77883200	1.00696800	−2.21517400
H	−3.72620600	1.64051700	0.81303600
H	−3.25164200	−0.07241600	2.27779700
H	1.50307600	−0.30972100	−2.13161600
H	3.71774400	−1.34348100	−1.86495600
H	4.05998300	0.37249500	2.04380000
H	1.82931500	1.40307100	1.78670600
H	6.04567600	−0.62397600	0.91331000
H	5.27646300	−2.20383300	0.76006400
H	5.87858500	−1.38196400	−0.68010100
H	−2.76540100	1.90810800	−1.37636800
O	−3.48667200	−2.56643000	0.03765400
O	−4.21755000	−1.66465900	−0.26236300

**Table:** Structure no.: Compound 9′ (PC′)

	X	Y	Z
C	−1.40426600	−1.04548000	1.44662100
C	−1.68555100	−1.82340900	0.18740800
C	−1.09274600	−1.21146500	−1.08908500
C	−1.04657200	0.32390900	−1.19275000
C	−2.26286200	1.11292300	−0.69113400
C	−3.03707800	0.42847800	0.44801200
C	−2.13304500	0.05367100	1.59173100
C	0.03562200	1.04323400	−0.40493900
N	−0.34863000	2.15063900	0.09390400
O	−1.70769500	2.34040900	−0.18489700
C	1.42004000	0.57601900	−0.22859400
C	2.08985700	−0.07613400	−1.26627400
C	3.40154100	−0.50244100	−1.09570100
C	4.07160300	−0.30329500	0.11273200
C	3.39268500	0.34800700	1.14672900
C	2.08453000	0.78172300	0.98376600
C	5.47822500	−0.80184400	0.31221800
H	−0.70141000	−1.40820700	2.18673200
H	−1.36161400	−2.86173500	0.27210200
H	−1.66786100	−1.60667200	−1.92951400
H	−0.07484700	−1.59067700	−1.19917600
H	−0.87264100	0.56617700	−2.24738300
H	−3.83020700	1.10160700	0.77808400
H	−2.08776800	0.69923500	2.45978400
H	1.59470500	−0.23388900	−2.21866100
H	3.91295700	−0.99690900	−1.91474000
H	3.89610500	0.51246600	2.09373700
H	1.56381200	1.27674100	1.79541200
H	6.06084900	−0.10257700	0.91408800
H	5.47227300	−1.76187200	0.83586200
H	5.98435400	−0.94550300	−0.64291400
H	−2.95919800	1.36969500	−1.48891500
O	−3.10850300	−1.95982100	0.08727000
O	−3.72892700	−0.69255500	−0.14190600

## Figures and Tables

**Table:** Gibbs free energies and relative ΔG energies (kcal/mol) of the reactant complexes, transition states, and products for both mechanisms with M06-2X/6-311+G(d,p) level in DCM.

Compound	M06-2X/6-311+G(d,p)	

	Gibbs free energies (au)	*ΔG* (kcal/mol)
**RC (7+ ** **^1^** **O** **_2_** **)**	−860.442697	0.0
**RC′ (7+ ** **^1^** **O** **_2_** **)**	−860.438132	2.9
**TS1**	−860.428228	9.1
**TS1′**	−860.429531	8.3
**9**	−860.528665	53.9
**9′**	−860.528323	53.7
