# Supplementary figures and images for: Synthesis and photooxygenation of 3-(p-substituted phenyl)-3a,8a-dihydro-4H-cyclohepta[d]isoxazoles: facial selectivity
Source: Turk J Chem. 2024 May 28;48(4):691–700. doi: 10.55730/1300-0527.3688 (PMC11407366; doi:10.55730/1300-0527.3688)

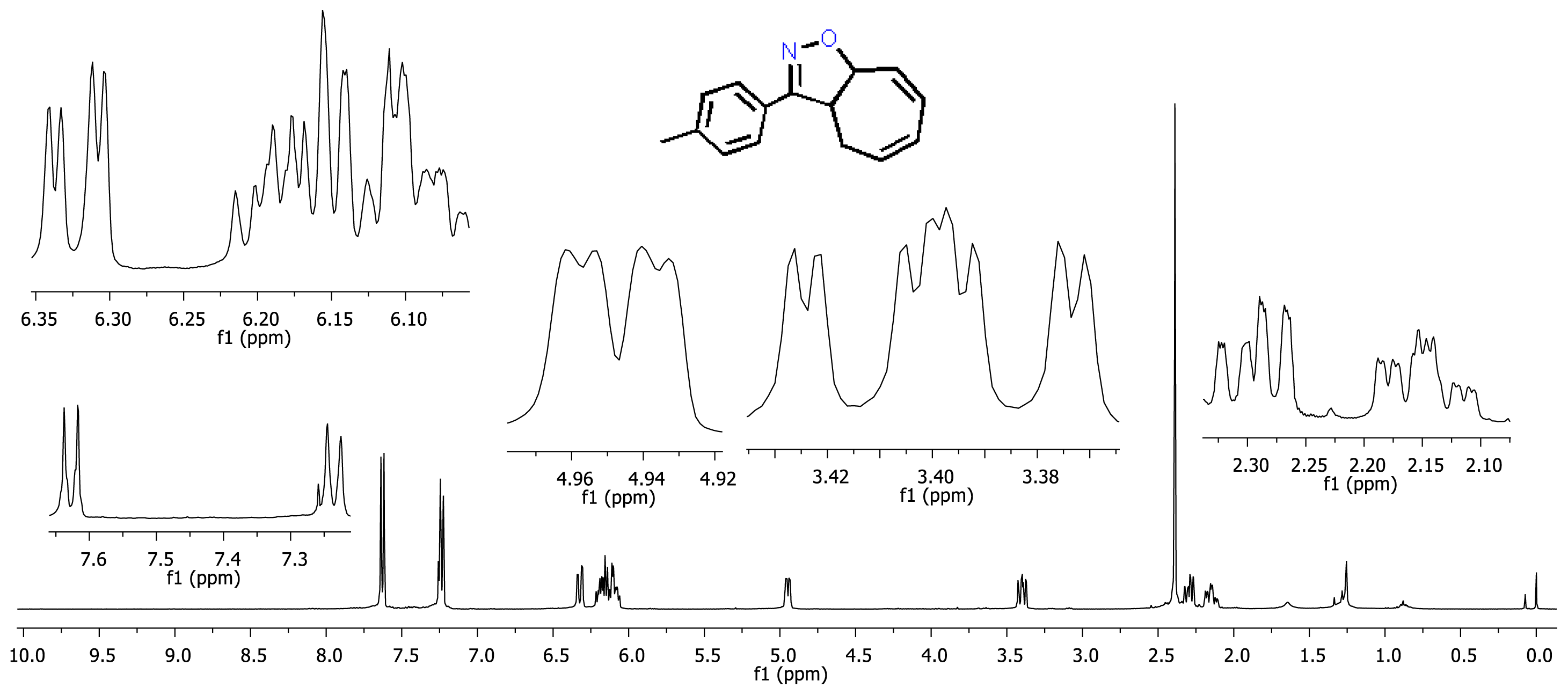

Supplement: Figure S1 — 1H-NMR spectrum of 7 (400 MHz, CDCl3). [file tjc-48-04-691s1.tif]

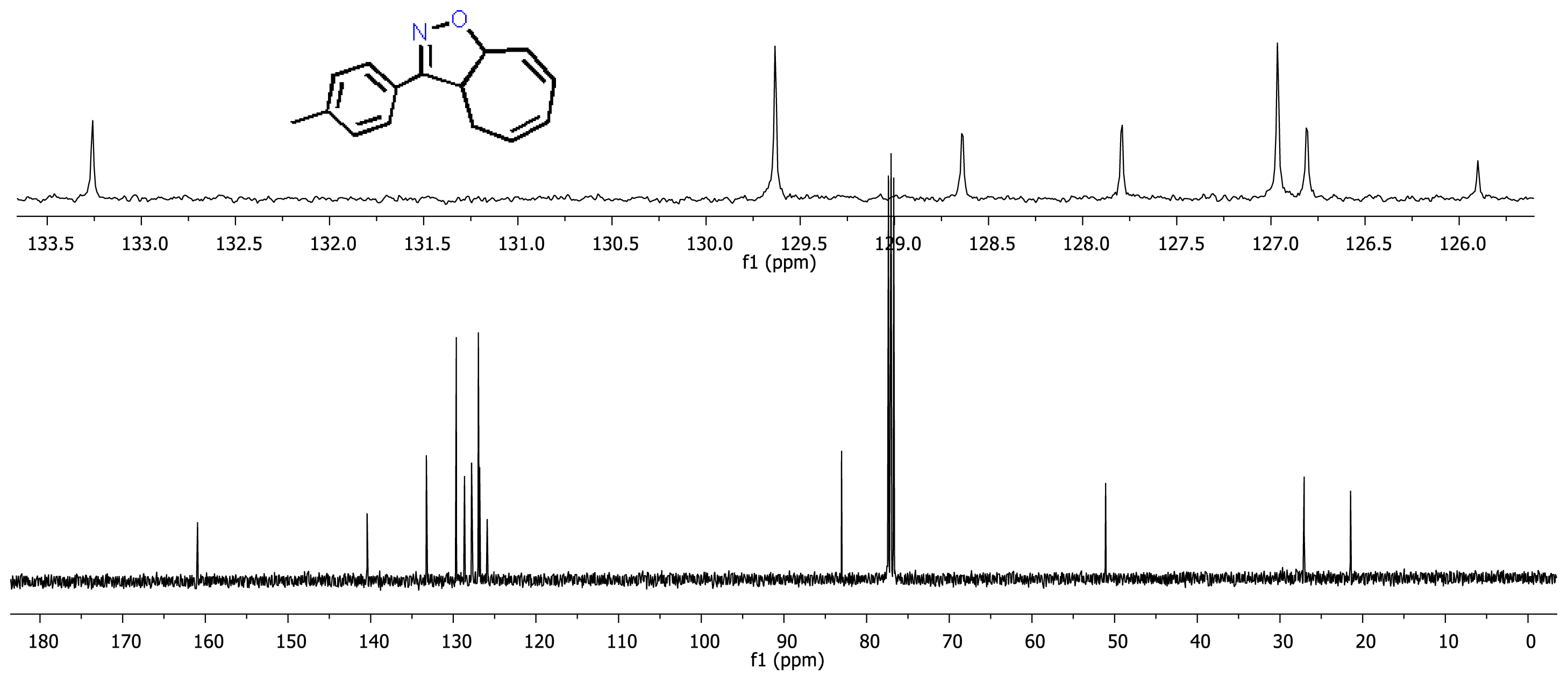

Supplement: Figure S2 — 13C-NMR spectrum of 7 (100 MHz, CDCl3). [file tjc-48-04-691s2.tif]

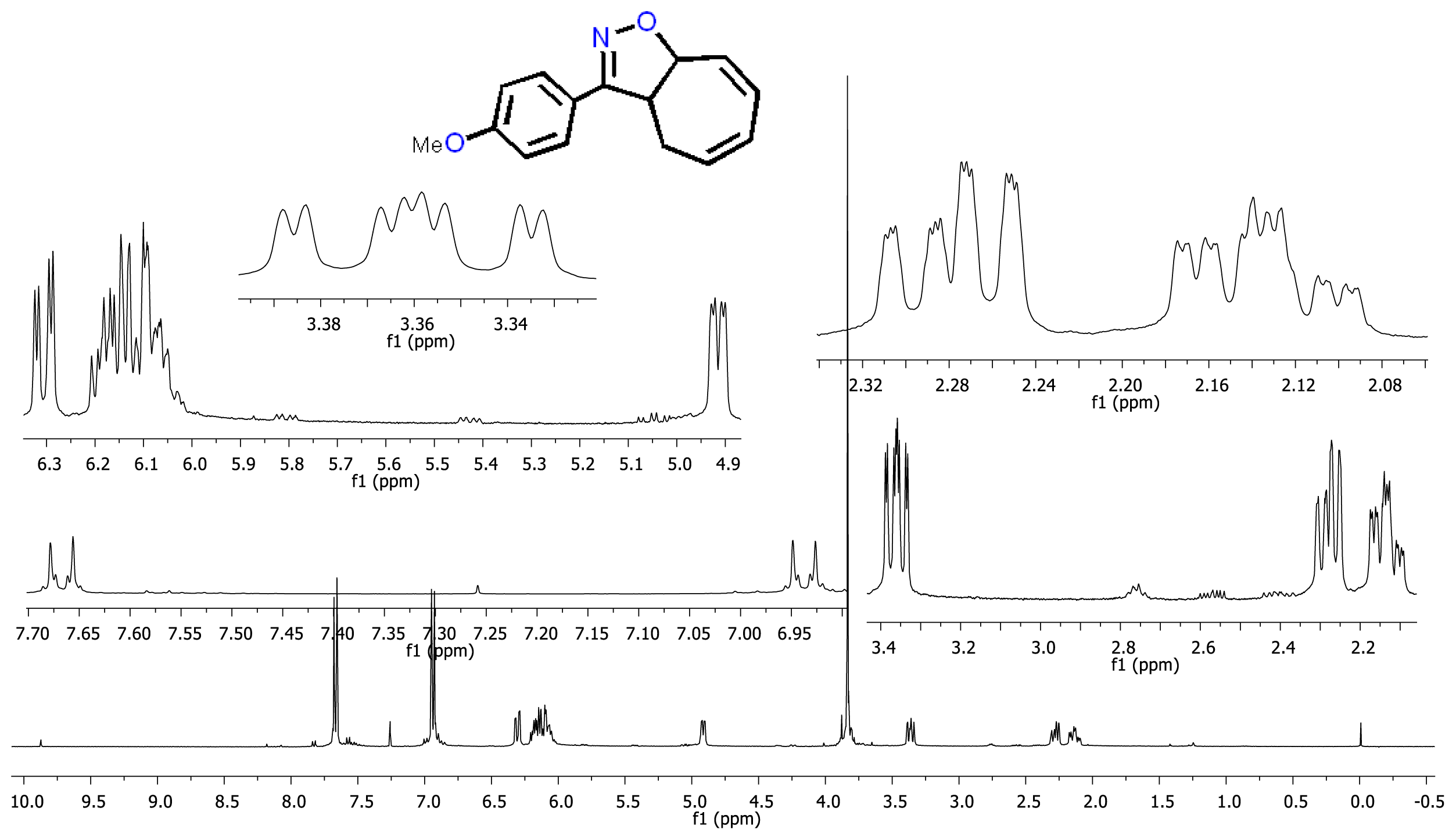

Supplement: Figure S3 — 1H-NMR spectrum of 8 (400 MHz, CDCl3). [file tjc-48-04-691s3.tif]

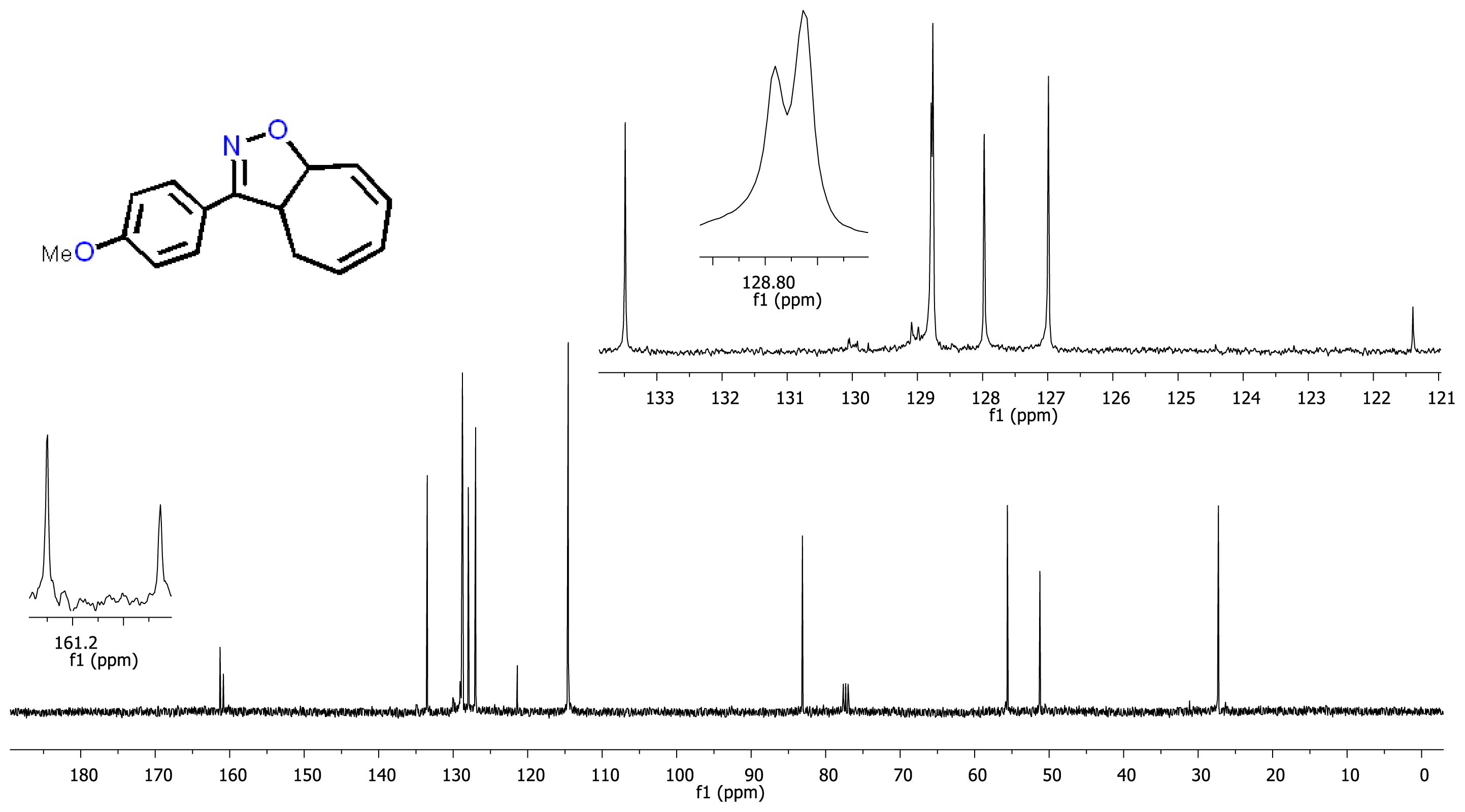

Supplement: Figure S4 — 13C-NMR Spectrum of 8 (100 MHz, CDCl3). [file tjc-48-04-691s4.tif]

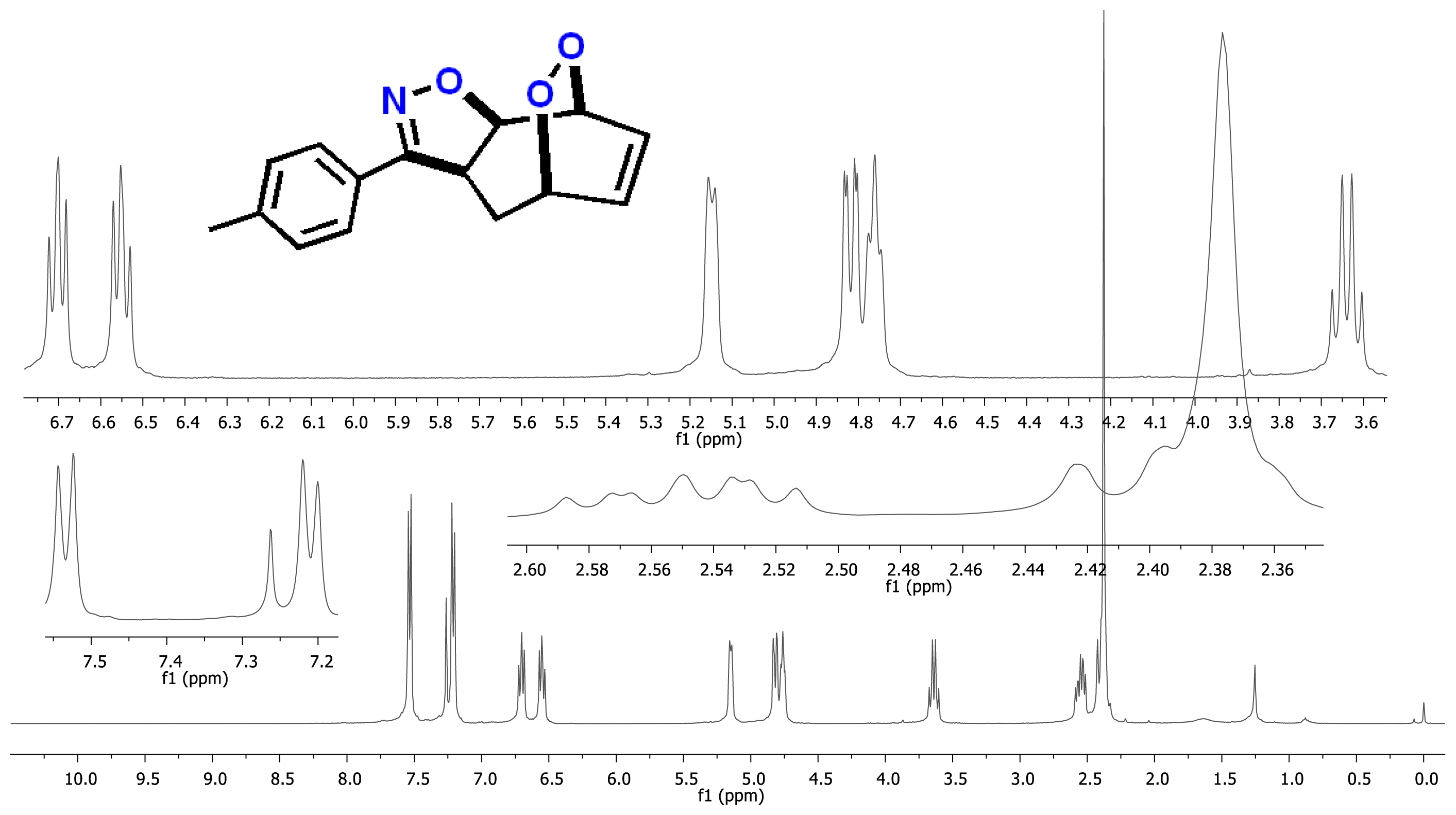

Supplement: Figure S5 — 1H-NMR spectrum of 9 (400 MHz, CDCl3). [file tjc-48-04-691s5.tif]

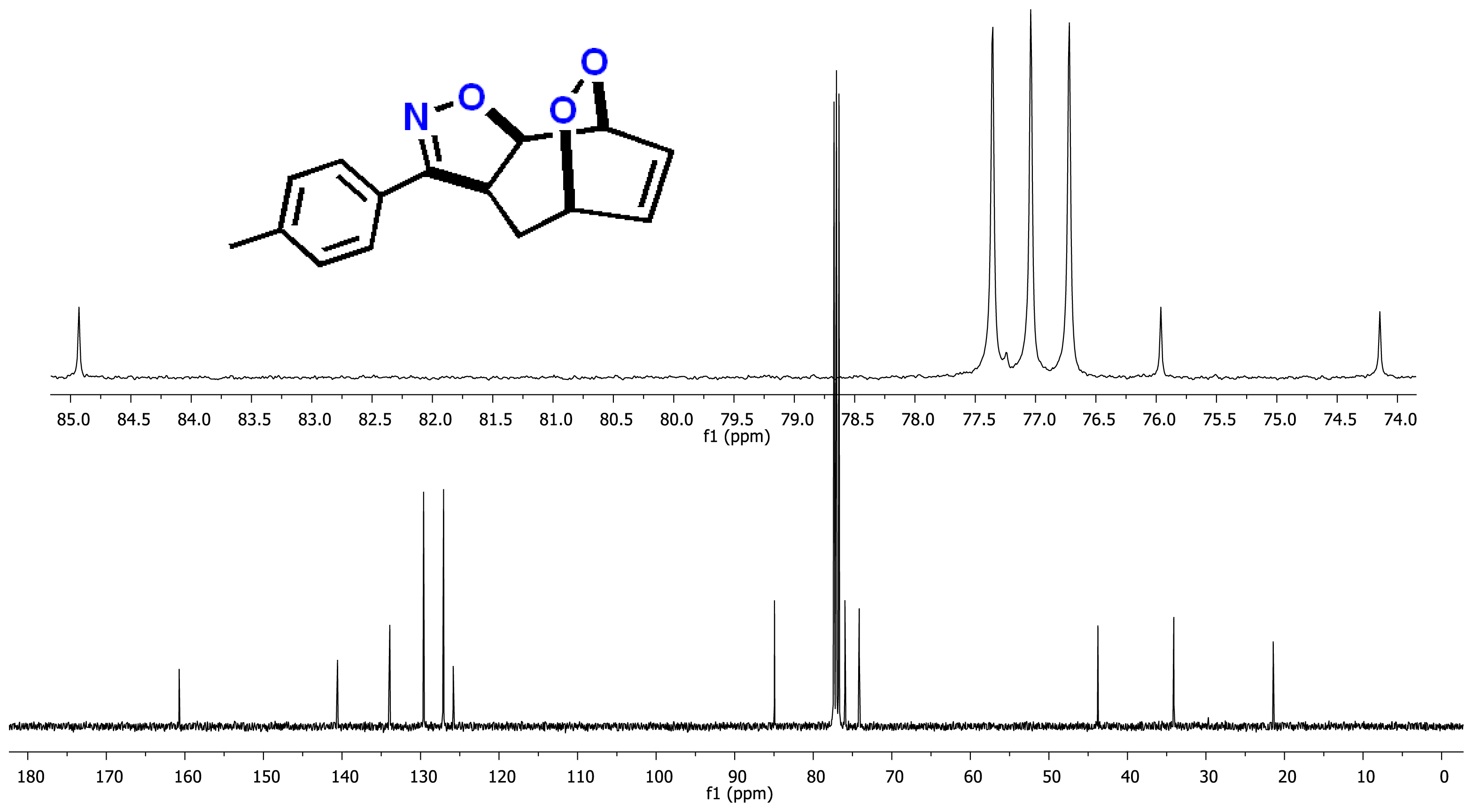

Supplement: Figure S6 — 13C-NMR spectrum of 9 (100 MHz, CDCl3). [file tjc-48-04-691s6.tif]

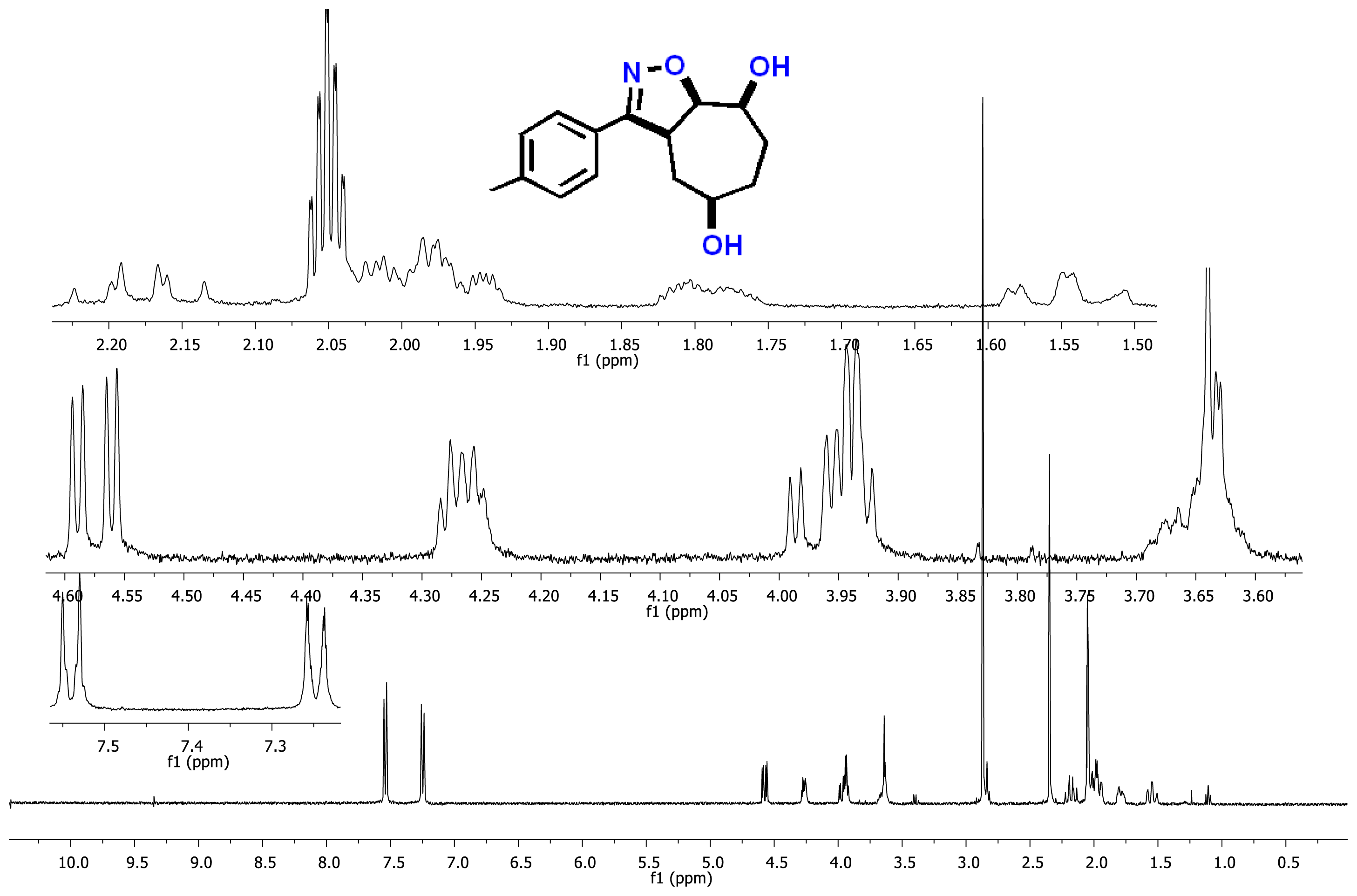

Supplement: Figure S7 — 1H-NMR spectrum of 10 [400 MHz, (CD)3CO)]. [file tjc-48-04-691s7.tif]

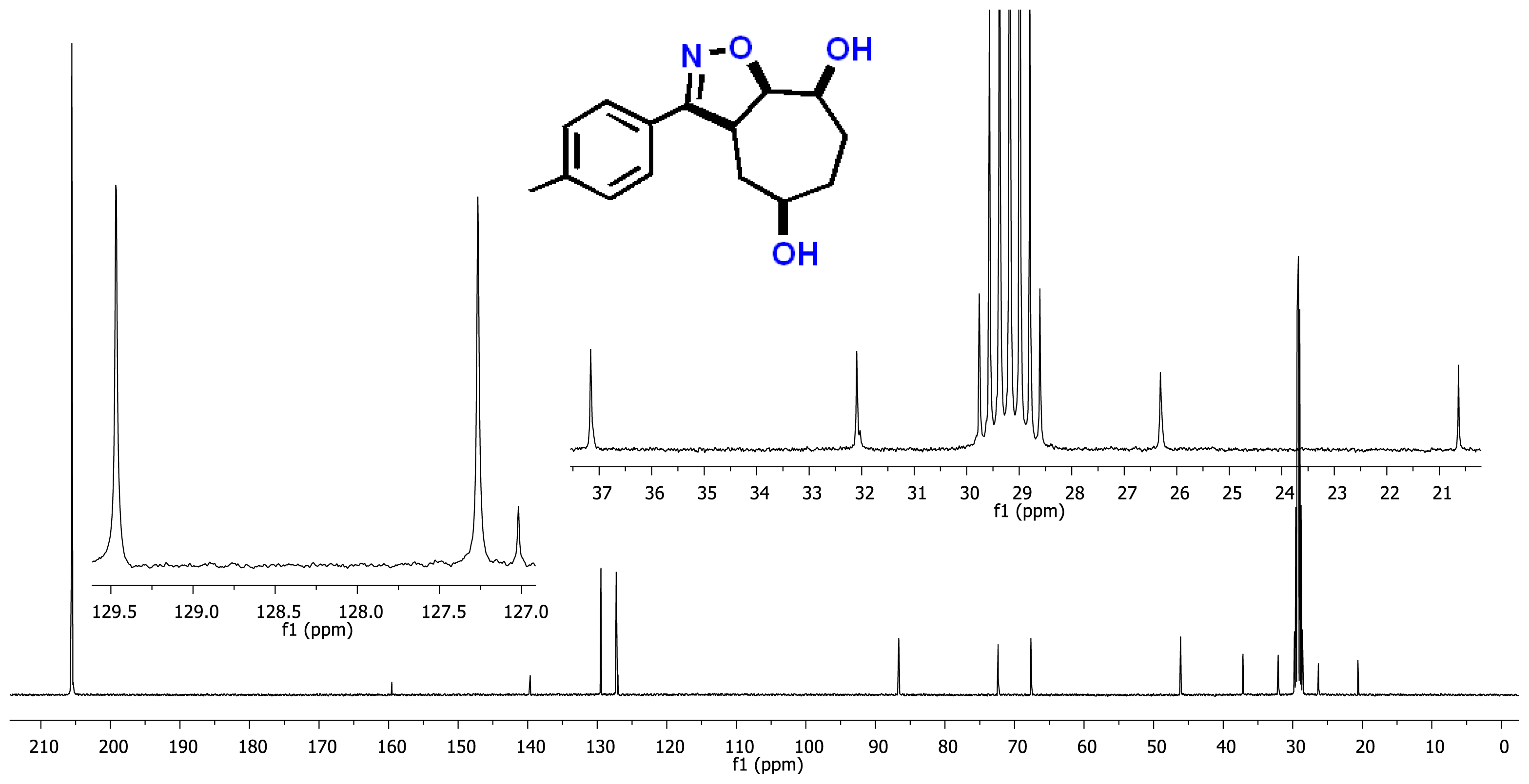

Supplement: Figure S8 — 13C-NMR spectrum of 10 [100 MHz, (CD)3CO)]. [file tjc-48-04-691s8.tif]

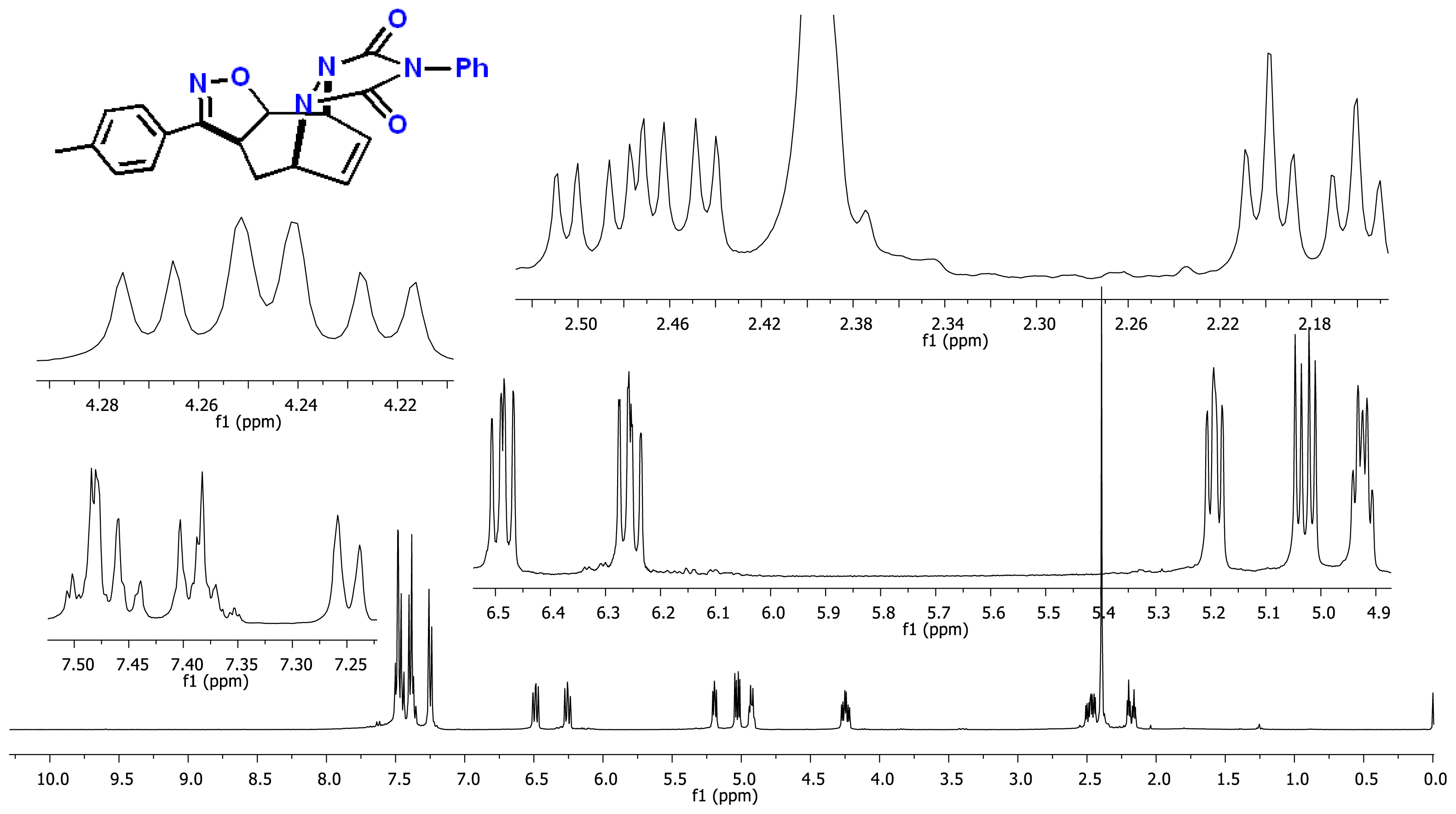

Supplement: Figure S9 — 1H-NMR spectrum of 11 (400 MHz, CDCl3). [file tjc-48-04-691s9.tif]

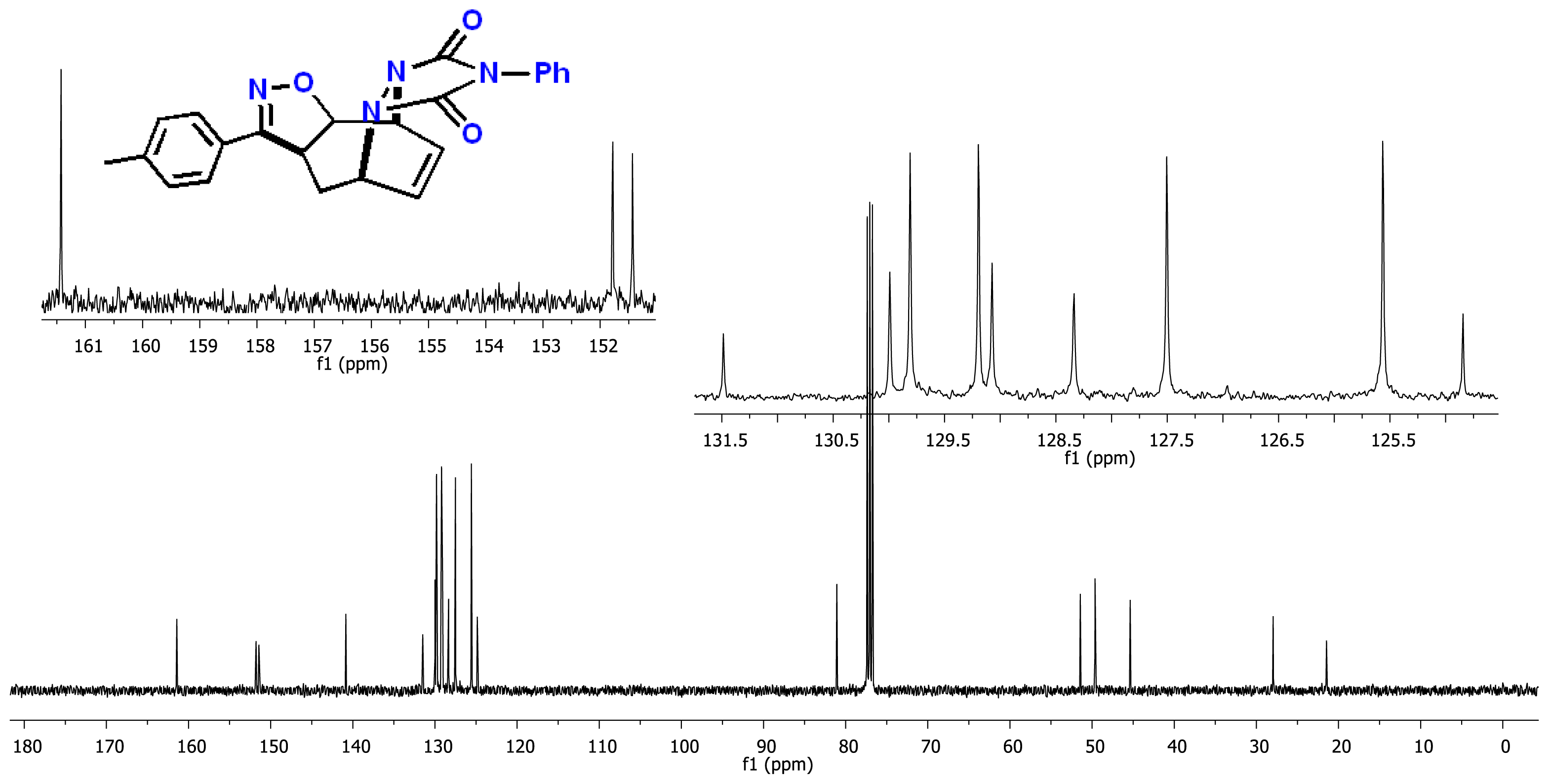

Supplement: Figure S10 — 13C-NMR spectrum of 11 (100 MHz, CDCl3). [file tjc-48-04-691s10.tif]

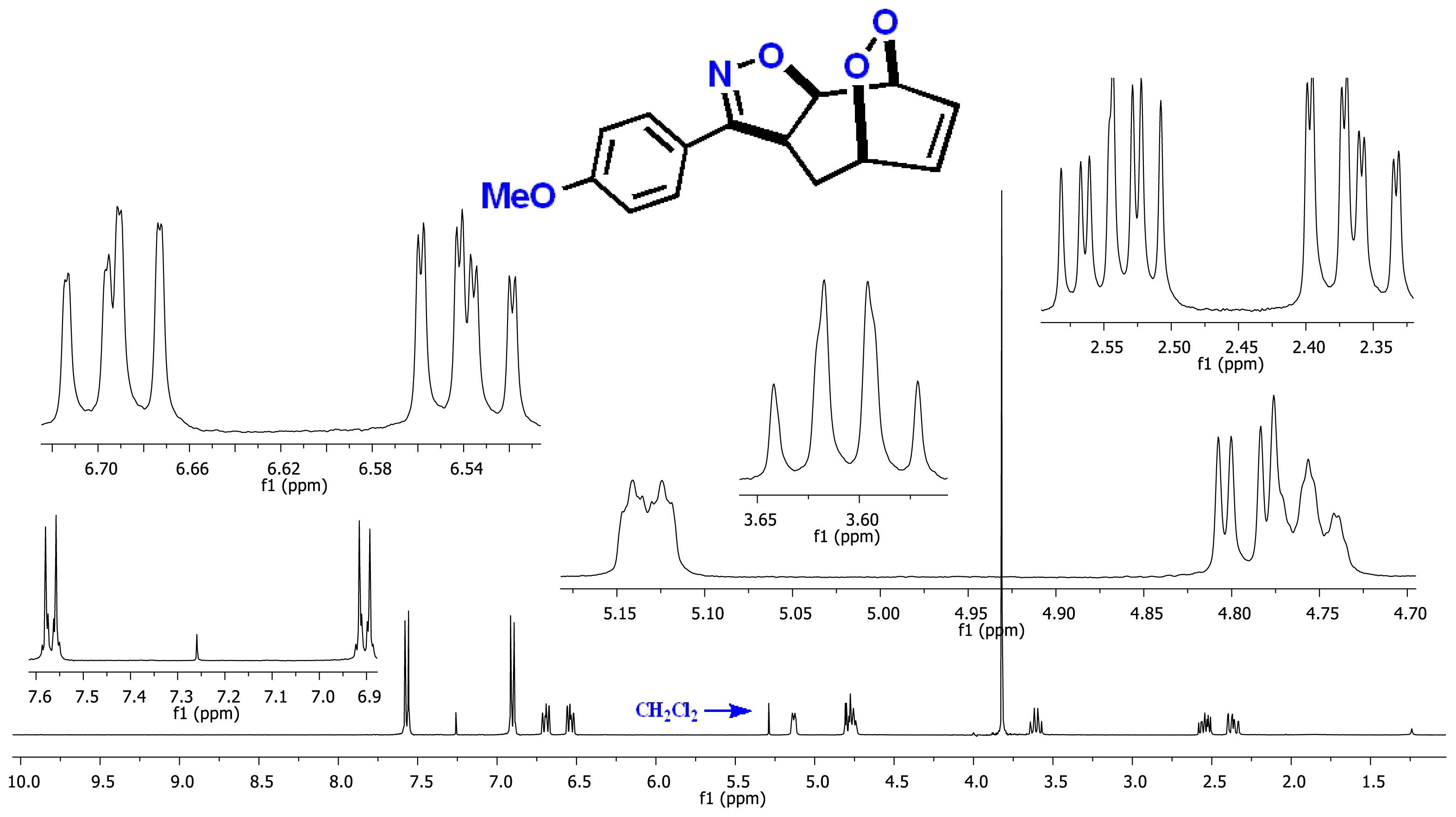

Supplement: Figure S11 — 1H-NMR spectrum of 12 (400 MHz, CDCl3). [file tjc-48-04-691s11.tif]

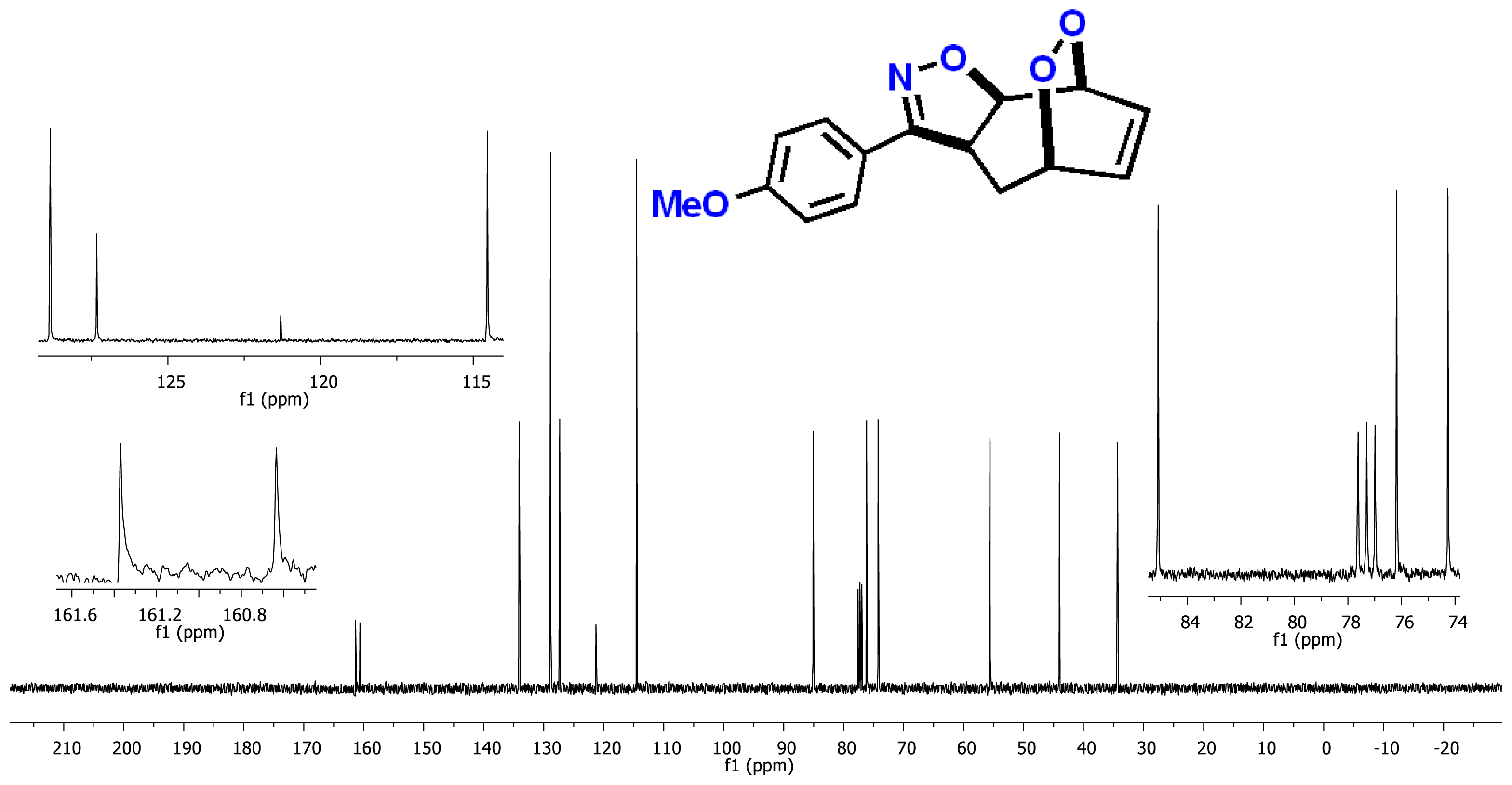

Supplement: Figure S12 — 13C-NMR spectrum of 12 (100 MHz, CDCl3). [file tjc-48-04-691s12.tif]

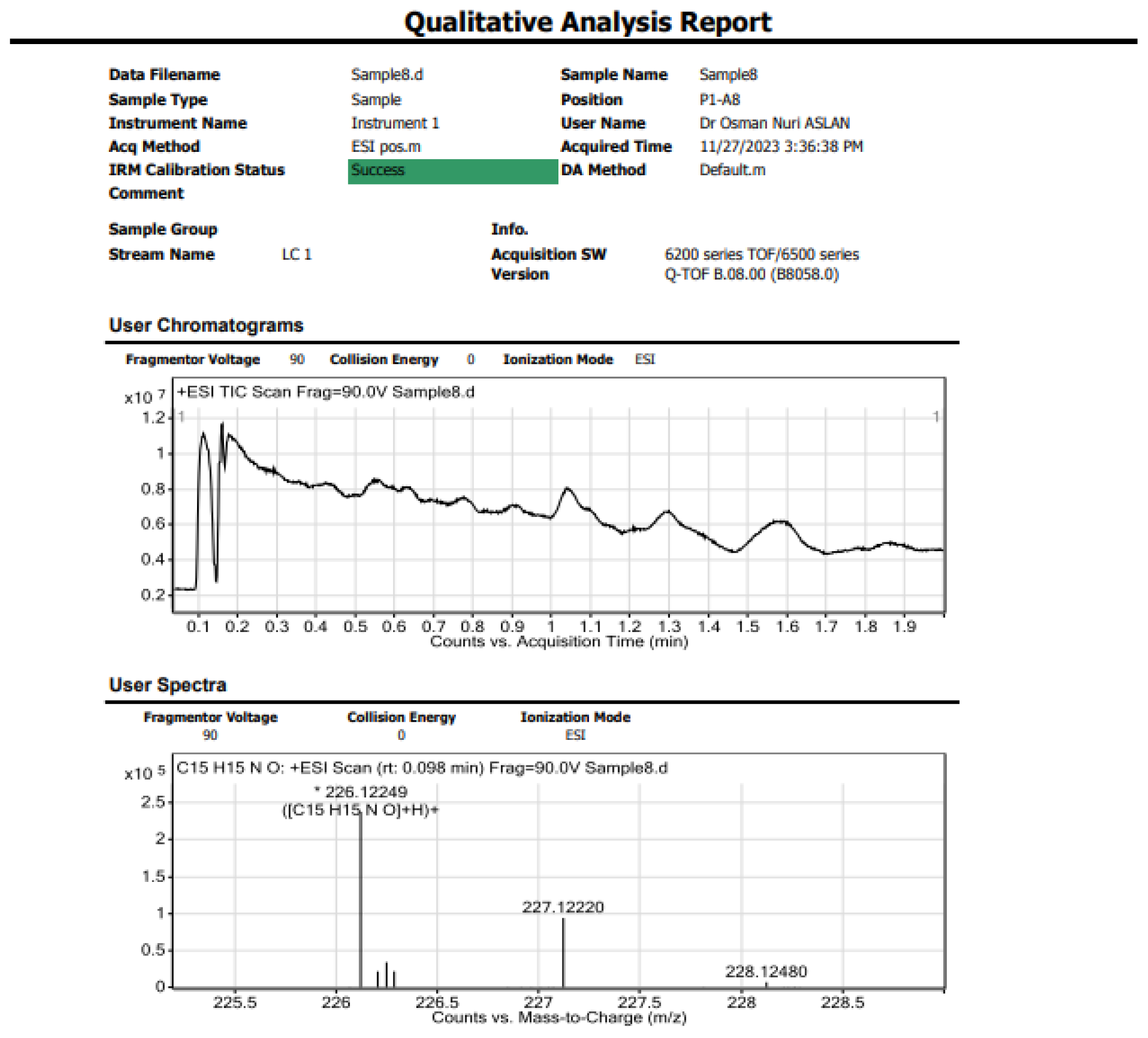

Supplement: Figure S13 — Compound 7. [file tjc-48-04-691s13.tif]

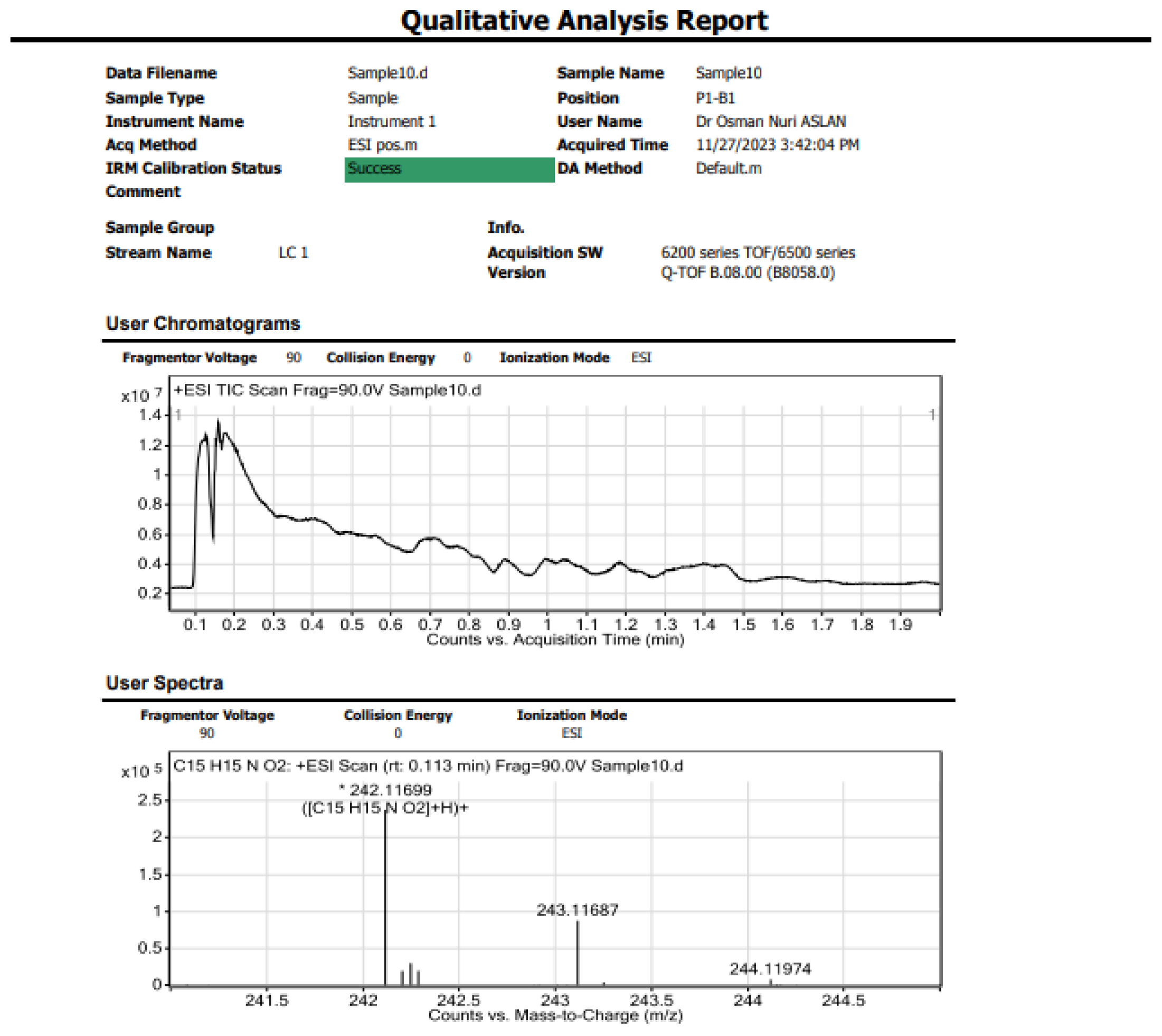

Supplement: Figure S14 — Compound 8. [file tjc-48-04-691s14.tif]

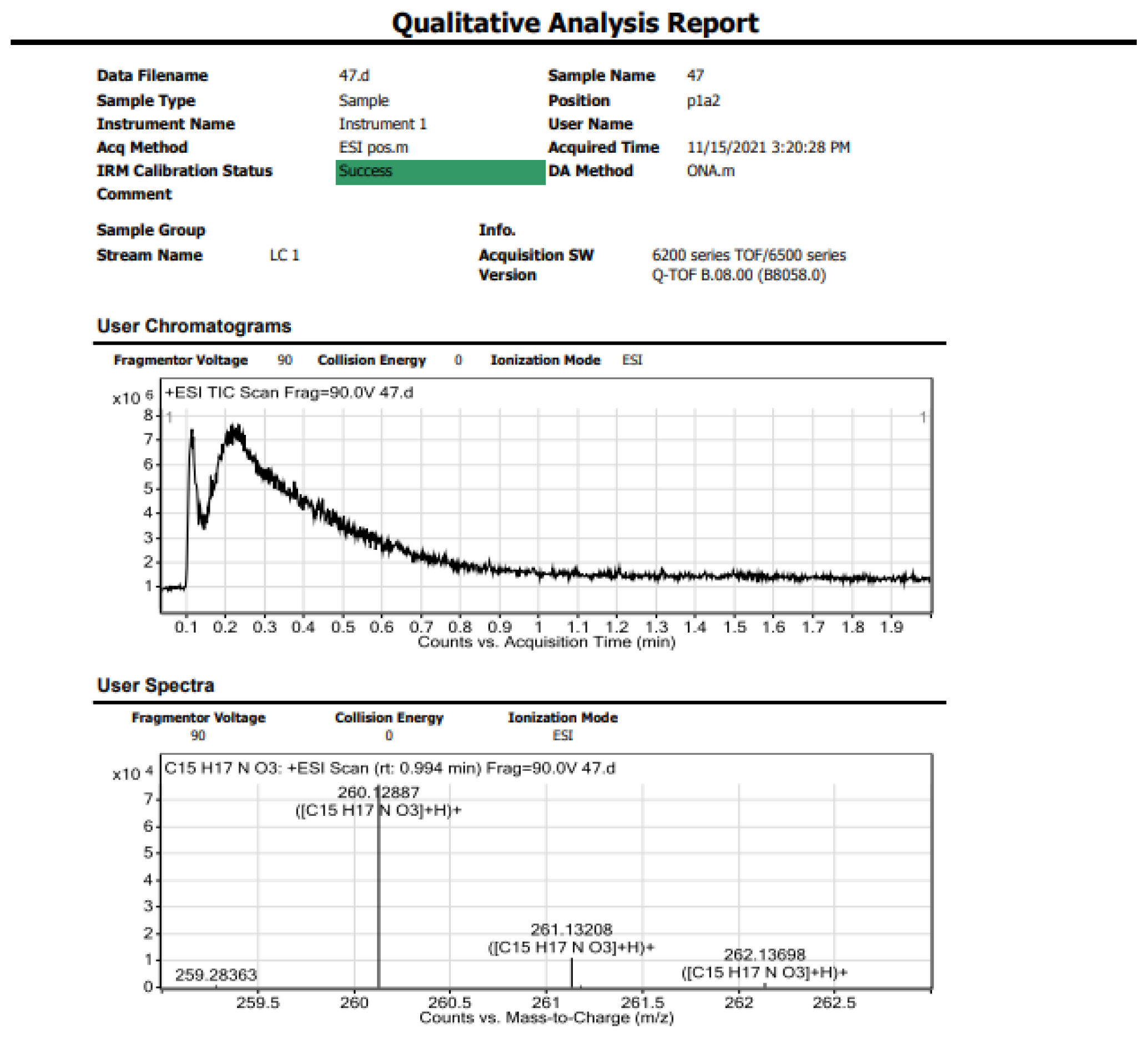

Supplement: Figure S15 — Compound 9. [file tjc-48-04-691s15.tif]

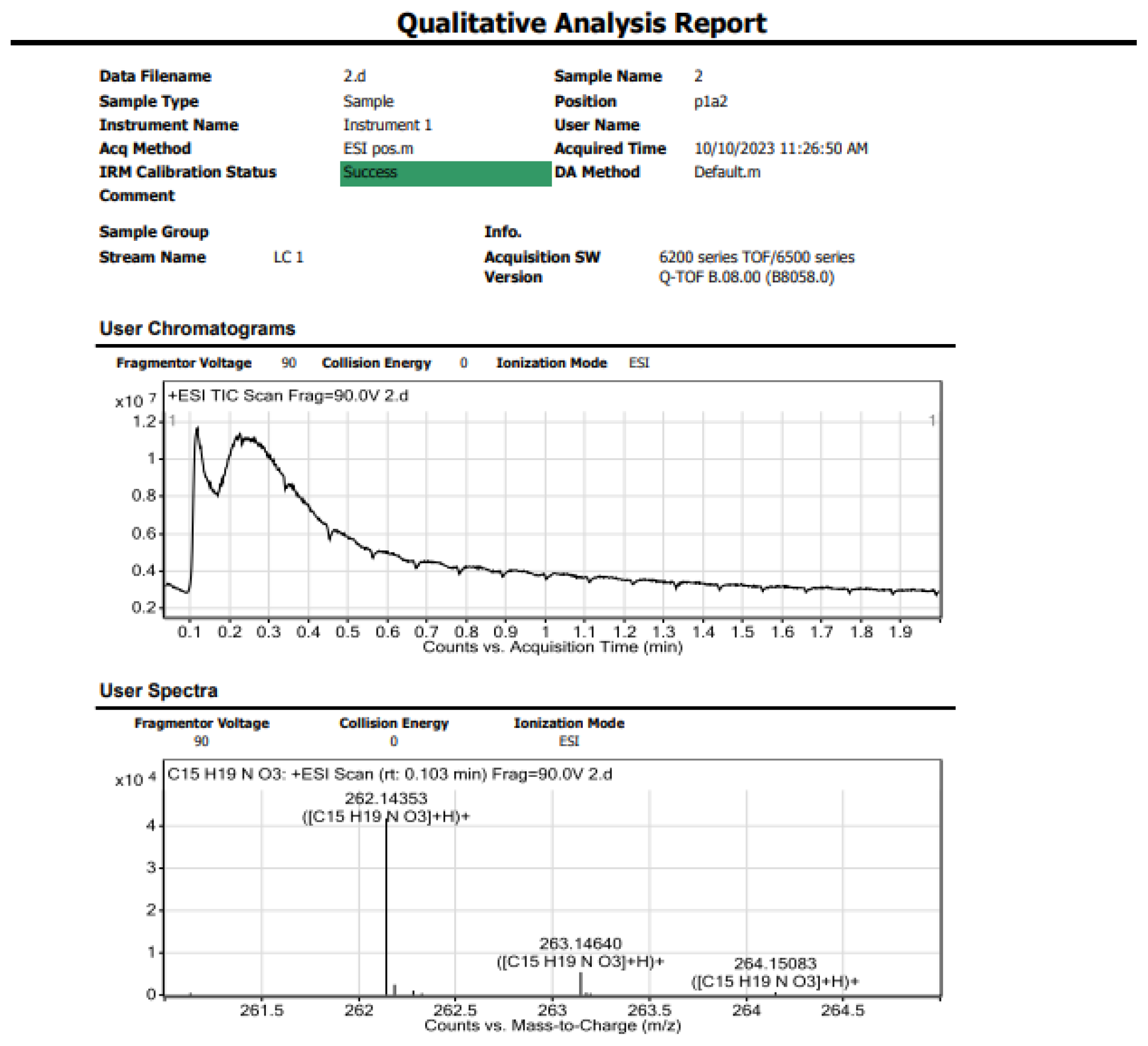

Supplement: Figure S16 — Compound 10. [file tjc-48-04-691s16.tif]

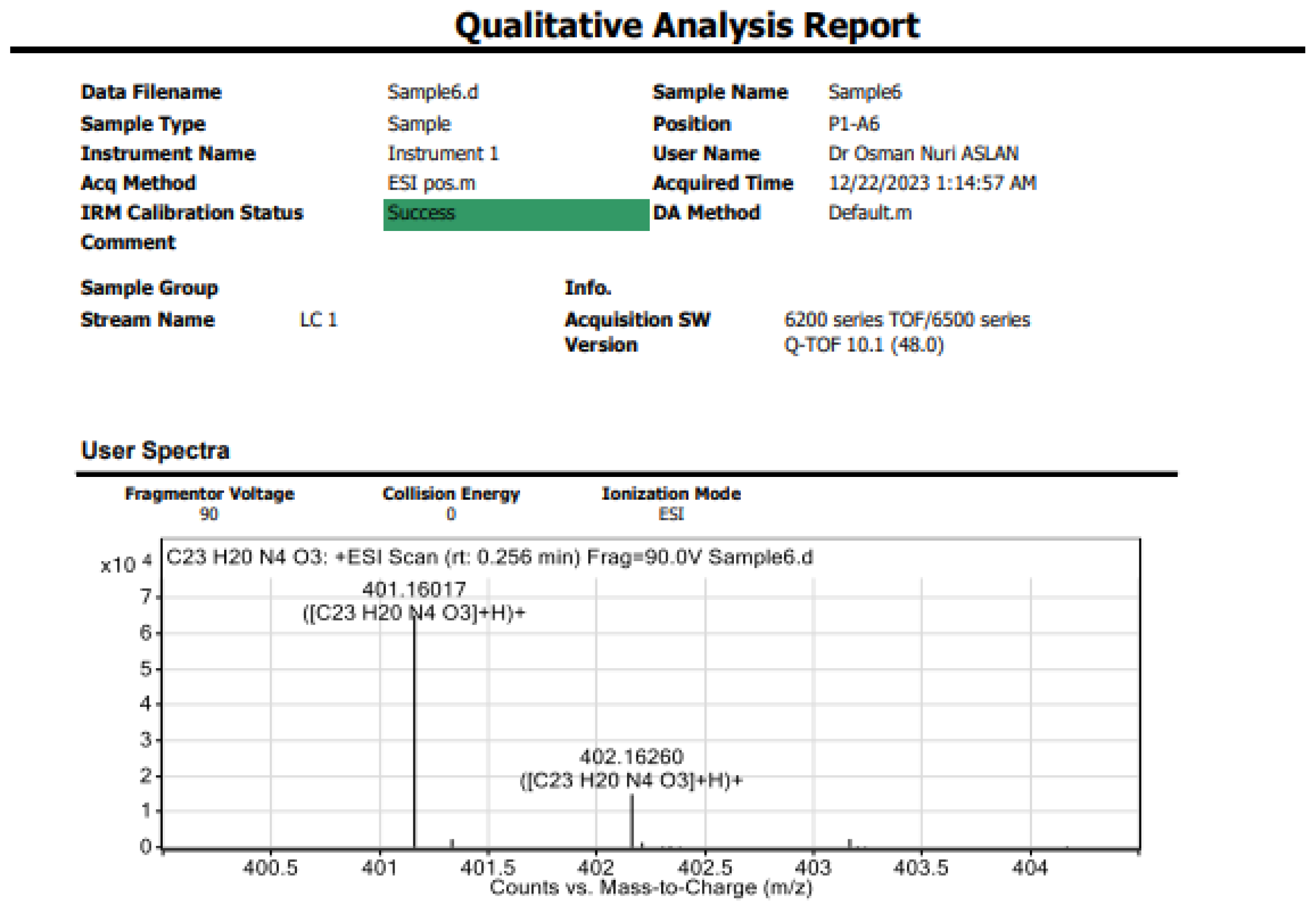

Supplement: Figure S17 — Compound 11. [file tjc-48-04-691s17.tif]

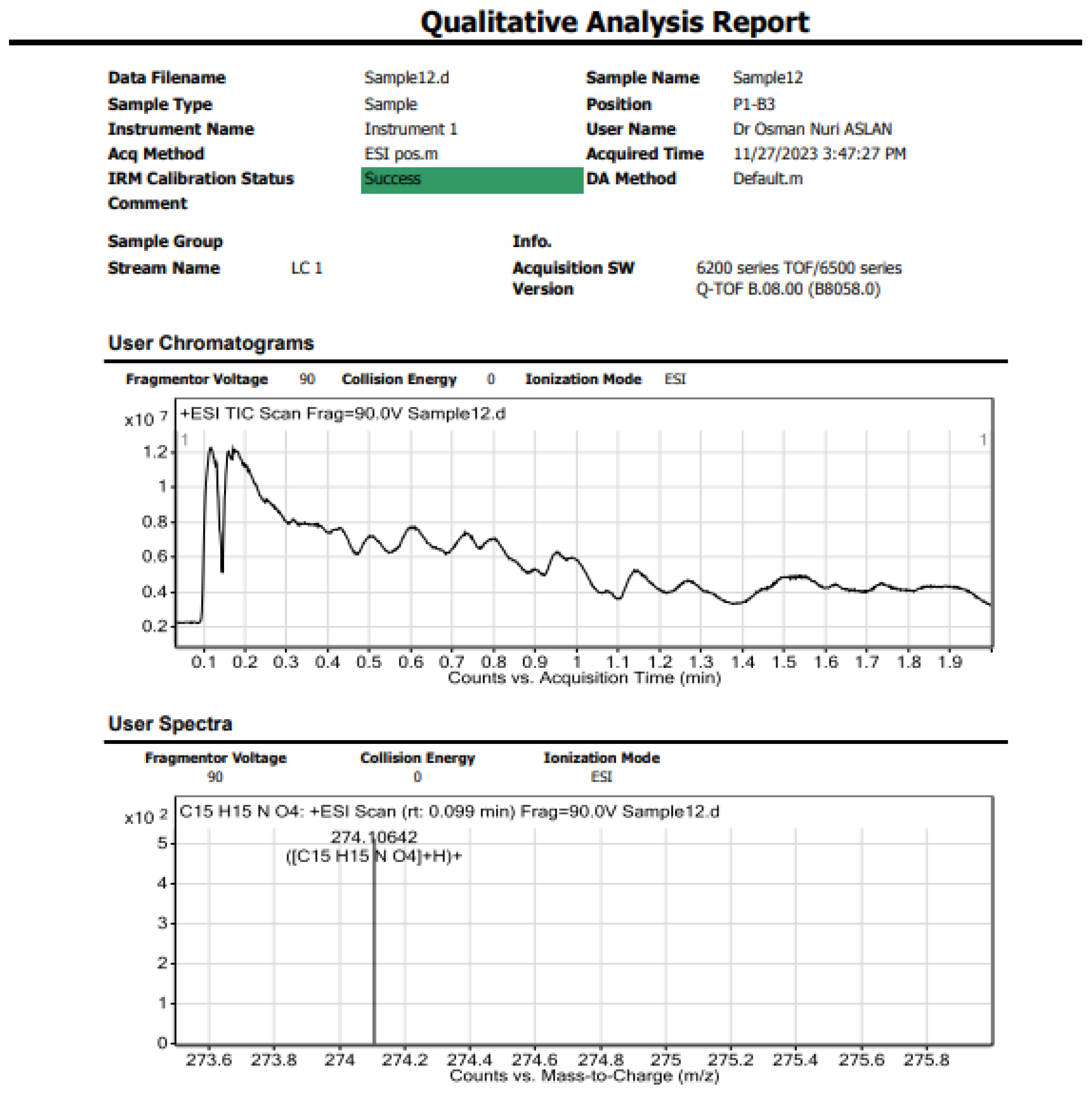

Supplement: Figure S18 — Compound 12. [file tjc-48-04-691s18.tif]
